# Crystal Structure of Melphalan Hydrochloride and Its Association with Caffeine Improves Its Antineoplastic Action

**DOI:** 10.1021/acsomega.5c01538

**Published:** 2025-05-15

**Authors:** Juliana Pereira da Silva, Carin Cristina da Silva Batista, Maria Lúcia Schumacher, Santiago Rodriguez, Alan Talevi, Paula Haddad, Guillermo Raul Castro, Fabio Furlan Ferreira

**Affiliations:** † Centro de Ciências Naturais e Humanas (CCNH), 74362 Universidade Federal Do ABC (UFABC), Av. Dos Estados, 5001, Santo André, São Paulo 09280-560, Brazil; ‡ Instituto de Ciências Ambientais, Químicas e Farmacêuticas, Departamento de Química, 505146 Universidade Federal de São Paulo, Rua São Nicolau, 210, Diadema, São Paulo 09913-030, Brazil; § Laboratorio de Investigación y Desarrollo de Bioactivos (LIDeB), Departamento de Ciencias Biológicas, Facultad de Ciencias Exactas, 28228 Universidad Nacional de La Plata (UNLP), Calle 47 y 115 (B1900AD), La Plata, Buenos Aires B1001, Argentina; ∥ 3Núcleo de Nanomedicina (NANOMED), Universidade Federal Do ABC (UFABC), Av. Dos Estados, 5001, Santo André, São Paulo 09280-560, Brazil

## Abstract

Melphalan hydrochloride (MEH) is a chemotherapy drug with antitumor activity, recognized for its classification as an alkylating agent. Over the past few decades, the drug has been administered to patients undergoing treatment for breast and ovarian cancers, and it is also intended for the treatment of multiple myeloma. It is commercially available in tablet and injection forms; however, its oral administration presents some limitations, including presystemic elimination and incomplete absorption. This study employs a simulated annealing approach and powder X-ray diffraction data to determine its crystal structure. The structure is confirmed by Rietveld refinement, which reveals good visual agreement between the generated model and experimental data. Given that MEH has low solubility in water, a screening conducted in the Mercury program (utilizing the CSD-Materials module) indicates the potential use of various molecular synthons to enhance the drug’s efficacy. Grinding processes (manual and mechanochemical) are conducted with MEH and a coformer, caffeine (CAF), to form stoichiometric mixtures. The vibrational characteristics associated with MEH and CAF show low energy levels. The effects on cell viability of the MEH-CAF combination are studied at different concentrations and reveal more significant cytotoxicity against the HeLa cell line (cervical tumor) compared to healthy MRC-5 cells (human fetal lung fibroblasts).

## Graphical abstract

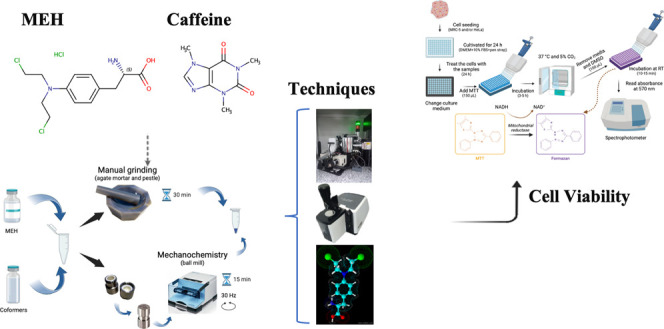

## 1 Introduction

Cancer is a disease characterized by abnormal cell growth with the potential to spread to other parts of the body, causing metastasis and possible tissue death. The cancer cycle begins when a normal cell undergoes mutation caused by genetic predisposition, exposure to chemicals, or failures in the natural process of DNA replication. Factors such as smoking, excessive sun exposure, radiation, certain viruses (such as HPV), poor diet, and family history can increase the risk of mutations that lead to cancer. Cancer cells ignore normal growth signals, becoming self-sufficient, and are insensitive to apoptosis signals, promoting their uncontrolled oversight.^1^

In 2022, about 20 million people worldwide were diagnosed with cancer, the most common being lung, breast, colorectal, prostate, skin, and stomach.^2^ According to the American Cancer Society, there were an estimated 2,001,140 new cancer cases and 611,720 cancer deaths in the U.S. in 2024.^3^ Breast (313,510) and prostate (299,010) are among the most prevalent cases of cancer, followed by lung and bronchus (234,580), colorectum (152,810), and melanoma of the skin (100,640). Although comprising lower estimated cases, multiple myeloma (35,780) and ovarian (19,680) are also of significant incidence. Anemia, hypercalcemia, renal failure, severe pathologic fractures, and recurring bacterial infections are the most frequent consequences of multiple myeloma.^4^ These issues must be handled concurrently with the therapy of the disease since the existence of one or more of them frequently serves as the first indicator of myeloma.^5^

The therapeutic approach differs based on the specific type of cancer, the manifestation of symptoms, and the promptness with which the patient seeks medical intervention. Although there are many treatment options, chemotherapy is the first choice in cancer treatment, where drug administration is the focus. The drugs used in this intervention reach the bloodstream and spread throughout all regions of the body, eradicating the malignant cells that make up the neoplasm and, at the same time, inhibiting its metastatic potential.^1^

Although several types of drugs are available for chemotherapy treatment, our focus is on melphalan hydrochloride (MEH). Bergel and Stock synthesized it in 1953 from phenylalanine, which subsequently led to the synthesis of several other compounds; however, based on their initial biological tests, “melphalan” (MEL) (L-*p*-bis­(2-chloroethyl)­amino- *l* -phenylalanine) was identified as the most promising compound in the series.^6^ Over the years, further pharmacological characterization of MEH confirmed its chemotherapeutic potential.

MEH is currently used to treat various types of cancer, mainly ovarian and breast cancer. However, its primary use is treating multiple myeloma (MM), for which it has received approval from the FDA (Food and Drug Administration, USA).^4,7^ Its chemical structure is shown in Figure 1A. MEH is commercially available in injectable and tablet dosage forms. However, its oral administration has some limitations: presystemic elimination, variable and incomplete absorption, and reduced bioavailability.^8^ In this context, we observed that its efficacy could be improved by increasing its solubility in water, so we explored the possibility of forming a cocrystal.

**Figure 1:**
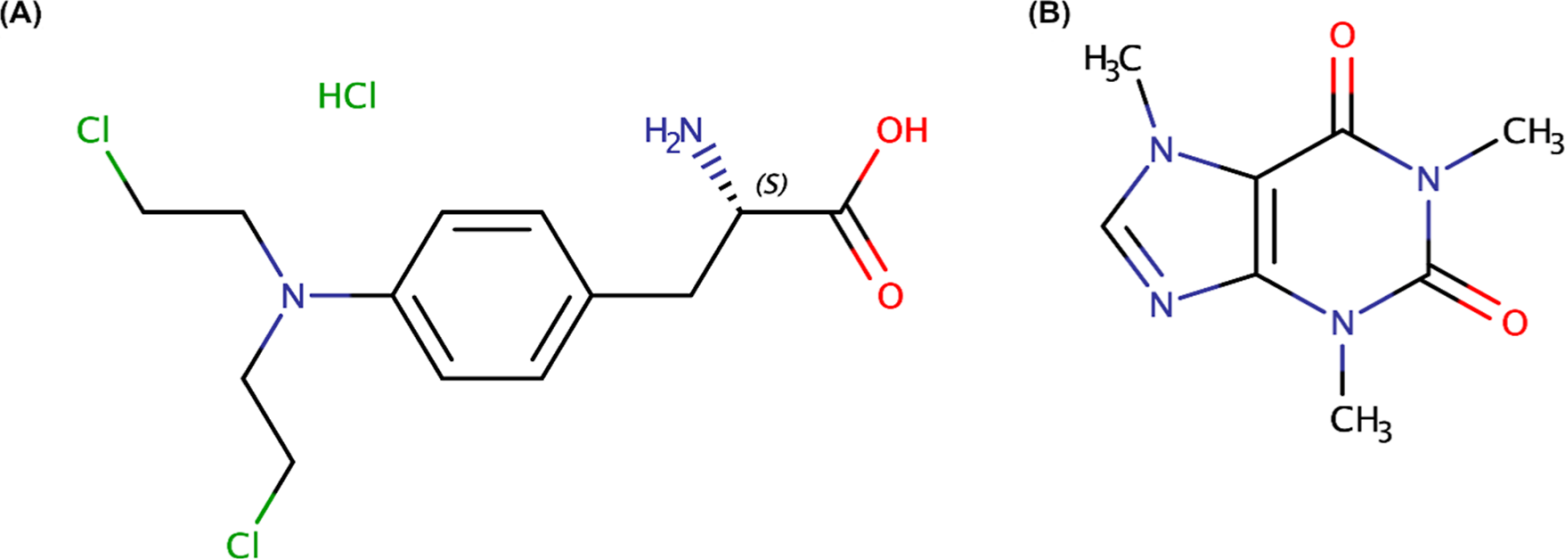
Chemical structure of (A) melphalan hydrochloride and (B) caffeine.

Pharmaceutical cocrystals are homogeneous materials formed by two or more compounds in stoichiometric proportions. In recent decades, they have stood out for being used to obtain new pharmaceutical forms.^9,10^ These cocrystals can comprise an API (active pharmaceutical ingredient), a nontoxic molecule, or another API. Thus, MEH is a drug that, when associated with its candidate coformers, can improve its solubility, especially in water. CAF (caffeine) is a candidate coformer that interacted better with MEH in the cocrystal preparation attempt.

Caffeine (CAF; Figure 1B) is a natural product of the purine alkaloid class and is present in products derived from plants such as grains (Coffea arabica and Paulinia cupana) and leaves (mate tea and black tea). CAF has been shown to enhance the effectiveness of chemotherapy in significant ways. First, it can stimulate vasodilation, which enables the drug to target the tumor site better and augments chemotherapeutic efficacy by fixing leaky blood vessels.^11^ Second, CAF can boost the amount of oxygen reaching the tumor, which is advantageous for chemotherapy and radiotherapy.^12^ Third, the literature reports that CAF inhibits homologous recombination (HR) factors, which help repair DNA lesions and are known to be overexpressed in certain types of cancer (such as colorectal cancer), making the cancerous cells highly resilient to DNA damage.^12^

In this study, we aim to determine the crystal structure of MEH and its association with CAF. By understanding the molecular interactions between these two compounds, we hope to provide insights into their synergistic effects in cancer treatment.

## 2 Materials and Methods

### 2.1 Samples

Farmabios SpA (Pavia, Italy) kindly donated the MEH sample, which was kept at low temperatures (4 °C), as received, before its use. CAF was purchased from Sigma-Aldrich Co (USA). MRC-5 cells were donated by Princeton University (Princeton, USA), and HeLa tumor cells were donated by the Federal University of Rio de Janeiro (Rio de Janeiro, Brazil). Dulbecco’s Modified Eagle Medium (DMEM), Pen-Strep antibiotics (penicillin and streptomycin −10,000 μg mL^–1^), trypsin, and fetal bovine serum (FBS) were purchased from Life Technologies. MTT salt (3-(4,5-dimethyl-2-thiazolyl)-2,5-diphenyl-2H-tetrazolium bromide) (ref. M6494) was purchased from Thermo Fisher Scientific, and DMSO (dimethyl sulfoxide) was purchased from Lab Synth and used for biological assays. The water was pretreated using the Milli-Q Plus System (Millipore Corporation).

Mixtures of MEH and CAF were performed using a Retsch MM400 mixer mill and an agate mortar and pestle. Table 1 displays the mechanochemically produced samples.

**Table 1:** Syntheses Carried out to Obtain Samples of Melphalan Hydrochloride (MEH) and Caffeine (CAF) with Different Molar Ratios and Different Methodologies (Ball Mill (BM) and Agate Mortar (AM))^b^

code	substances	molar ratio (mmol)	method	solvent	synthesis time (min)
M1C1_BM25 μL	MEH-CAF	1:1	ball mill	milli-Q water 25 μL	15^a^
M1C1_BM0 μL	MEH-CAF	1:1	ball mill		15^a^
M1C2_BM25 μL	MEH-CAF	1:2	ball mill	milli-Q water 25 μL	15^a^
M1C2_BM0 μL	MEH-CAF	1:2	ball mill		15^a^
M2C1_BM25 μL	MEH-CAF	2:1	ball mill	Milli-Q water25 μL	15^a^
M2C1_BM0 μL	MEH-CAF	2:1	ball mill		15^a^
M1C1_AM25 μL	MEH-CAF	1:1	agate mortar	Milli-Q water 25 μL	30
M1C1_AM0 μL	MEH-CAF	1:1	agate mortar		30
M1C2_AM25 μL	MEH-CAF	1:2	agate mortar	Milli-Q water 25 μL	30
M1C2_AM0 μL	MEH-CAF	1:2	agate mortar		30
M2C1_AM25 μL	MEH-CAF	2:1	agate mortar	Milli-Q water 25 μL	30
M2C1_AM0 μL	MEH-CAF	2:1	agate mortar		30

a Processed inside 2 mL Eppendorf tubes with two 5 mm-diameter agate balls.

b The frequency used in the ball mill was 25 Hz.

### 2.2 Powder X-ray Diffraction (PXRD)

Powder X-ray diffraction (PXRD) data were collected in transmission geometry on a STADI-P (Stoe, Darmstadt, Germany) diffractometer operating at 40 kV and 40 mA, using Cu*K*α_1_ radiation (λ = 1.54056 Å). The powdered sample was deposited between two acetate cellulose foils, and the sample holder kept spinning during data collection. The integrated intensities were recorded by a Mythen 1K (Dectris, Baden, Switzerland) detector from 2.000 to 80.735° (2θ), in steps of 0.015° and a counting time of 300 s at each 1.05°.

### 2.3 Fourier Transform Infrared Spectroscopy

Fourier transform infrared spectroscopy (FTIR) measurements were carried out on Agilent Cary 630 equipment, between 4000 and 400 cm^–1^, with a resolution of 4 cm^–1^, in attenuated reflectance (ATR) mode. An average of 512 scans were used for the FTIR measurements.

### 2.4 Molecular Dynamics (MD) Simulations

To predict the potential interactions between caffeine and melphalan, a 300 ns molecular dynamics (MD) simulation in explicit water was carried out using the software GROMACS 2020.4.^13–19^ CHARMM36-jul22 was the selected force field for this purpose,^20^ and both compounds were parametrized using the SwissParam web server.^21,22^ The starting coordinates of both molecules were set randomly in a 14.92 nm^3^ dodecahedral box, which was solvated with TIP3P water.

The system underwent energy minimization using the steepest descent algorithm, employing a step size of 0.01 and ensuring a maximum force below 10 kJ mol^–1^ over a maximum of 50000 steps. Following this, a system equilibration phase was conducted under *NVT* ensemble conditions for 100 ps, applying position restraints at 293 K. The equilibration utilized the Berendsen-modified thermostat^23^ with a τ of 0.1 ps and a time step (dt) of 2 fs.

Subsequent pressure equilibration was carried out through a 100 ps *NPT* ensemble, maintaining position restraints and employing the Parrinello–Rahman barostat^24^ at 1 bar, with τ set at 2 ps and dt at 2 fs. Finally, a 300 ns MD simulation was conducted without position restraints, replicating the conditions of the *NPT* ensemble.

### 2.5 Biological Assays—Cell Viability

Cell viability was evaluated using an MTT assay to verify the interaction of the MEH-CAF (melphalan hydrochloride-caffeine) compound with eukaryotic cells. According to ISO 10993–5:2009,^25^ a system is viable, that is, nontoxic when it contains 70% of living cells at the end of the experiment. The cells were maintained until a confluence of 90% and passed 1:3 by trypsinization.

Cell viability was evaluated using the MTT colorimetric assay, which is based on the ability of viable cells to reduce MTT salt metabolically using the succinic mitochondrial dehydrogenase enzyme. The salt reduction promotes the formation of blue-purple formazan crystals, which accumulate in the cellular cytoplasm. The cells were plated at 10,000 cells per well in 96-well plates and incubated 24 h before any experiment. After this period, the cells were treated with the MEH-CAF compound in different concentrations and again incubated for 24 h. After this period, the cells were washed with PBS 1× (saline phosphate buffer), and 100 μL of MTT solution at a concentration of 0.1 mg mL^–1^ was added. The plates were maintained for 4 h under light protection in a 37 °C and 5% CO_2_ humid atmosphere. Subsequently, 150 μL of dimethylsulfoxide (DMSO) was added to dissolve the formazan crystals, and the plates were kept under agitation for 15 min for total dissolution. Absorption measurements were performed at λ = 570 nm in an automatic microplate reader (SynergyTM Multimode Microplate Reader Biotek).^26^

## 3 Results and Discussion

### 3.1 Crystal Structure Determination

The powder pattern indexing was carried out with an iterative use of singular value decomposition^27^ using TOPAS-Academic v7 (TA-7).^28^ The first 25 reflections were automatically selected using the graphical user interface (GUI), and the peak positions and peak areas were copied and pasted into an input file in jEdit*.* ^29^ A Split-Pearson VII peak shape function was chosen to fit the peaks, conducted without considering any peak shifts and Lorentz-polarization correction effects, and the crystallite size was restrained to have the same value for the selected reflections. The background was modeled using a 12th-order Chebyshev polynomial. The refined peak positions and peak areas were used in the indexing procedure in TA-7, considering all possible crystal systems. After analyzing systematic absences, the *P*2_1_ space group (monoclinic crystal system) was found. Then, a Pawley refinement^30^ was performed to extract the integrated intensities. The fundamental parameters approach^31^ was used to model the peak shapes. A full axial model^32,33^ accounted for the asymmetric peak shapes at low diffraction angles. The refined unit cell parameters were *a* = 5.20127 Å, *b* = 7.25908 Å, *c* = 21.16343 Å, β = 93.8203°, and V = 797.281 Å^3^. We then inserted these pieces of information into DASH,^34^ which uses a simulated annealing (SA) approach to solve crystal structures. It is worth mentioning that all steps in the structure determination procedure can be performed using DASH (peak fitting, indexing, Pawley refinement to extract intensities, space group determination, setting up the structural model, monitoring the structure solution progress, examination of the output structure, and Rietveld refinement). It requires a 3D model of the melphalan hydrochloride chemical structure, which was drawn using MarvinSketch 23.4.0–6250.^35^ In the SA approach, the full range of possible values of molecular positions and orientations and any flexible torsion angles (3 describing the positional coordinates, 4, of which three are independent, describing the molecular orientation, and 5 flexible torsion angles) were allowed to vary. Twenty-five runs (totaling 5 × 10^8^ movements) were globally optimized, and the best results were considered in the final Rietveld refinement (using TA-7); DASH generated the input file for TA-7.

After determining the crystal structure, we used TA-7, keeping the same approach for refining the background, unit cell parameters, and peak shapes as we used for the Pawley fit. We refined the atomic coordinates by considering restraints on bond distances and angles—using the Mogul geometry check tool^36,37^ —until convergence. We then inserted the hydrogen atoms in calculated positions using the Mercury software.^36^ They were also refined, maintaining the restraint conditions. Figure 2 shows the Rietveld plot of MEH. Table 2 shows the crystal data and details of the structure determination process. Atomic coordinates, bond lengths and angles, and hydrogen-bonding information are displayed in the Supporting Information material. More detailed information about the crystal structure determination procedure and refinement can be found elsewhere.^38–42^

**Figure 2:**
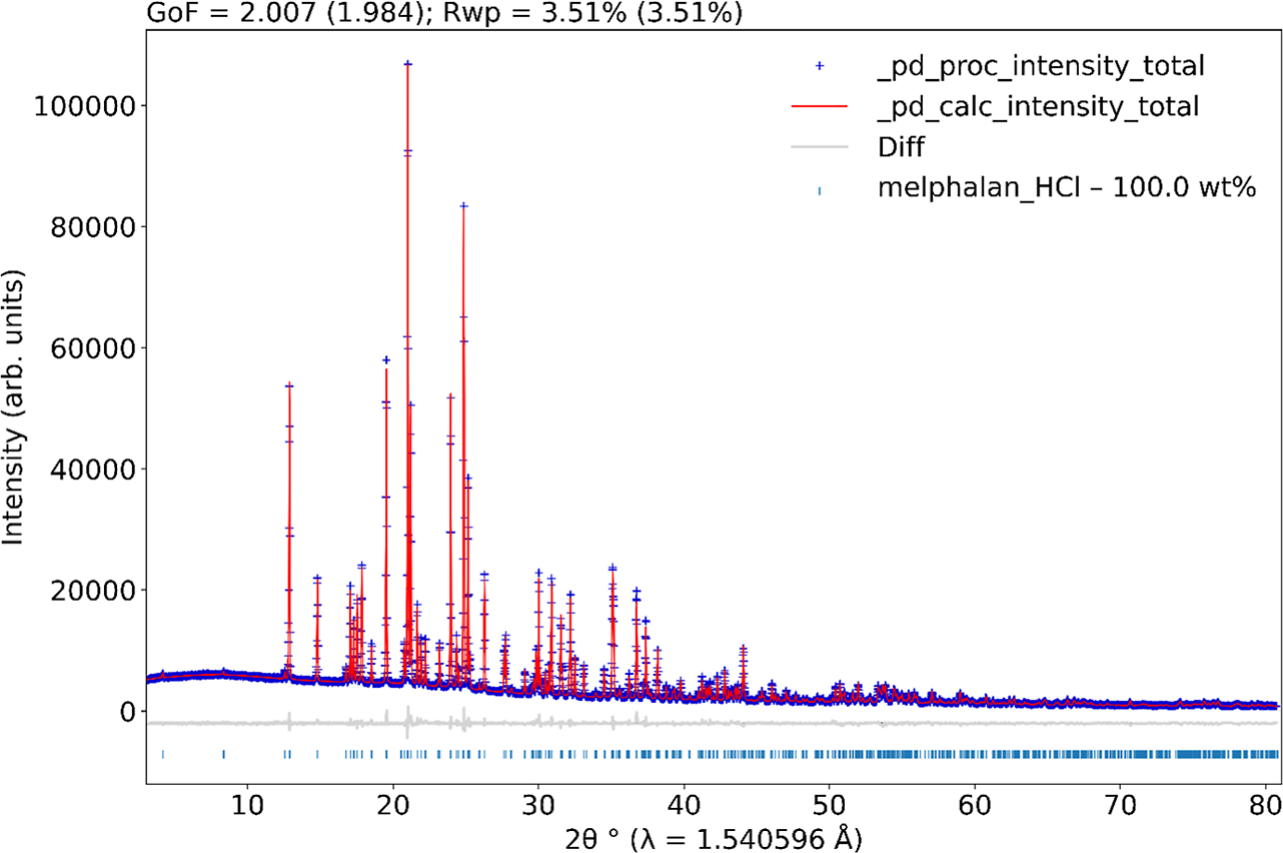
Rietveld plot of the melphalan hydrochloride sample. The blue crosses indicate the observed pattern, while the red line represents the calculated one. The gray line at the bottom displays the difference between the experimental and calculated patterns. The blue vertical bars stand for the Bragg reflections. The figure was generated using pdCIFplotter.^43^

**Table 2:** Crystal Data of MEH and Details of the Structure Determination Process

chemical formula	C_13_H_19_Cl_2_N_2_O_2_,Cl	
formula weight (g mol^–1^)	341.66	
crystal system	monoclinic	
Space group	*P*2_1_ (Nr. 4)	
*a*, *b*, *c* (Å)	5.20000(11), 7.26011(17), 21.1780(6)	
β (°)	93.8390(15)	
volume (Å^3^)	797.73(3)	
*Z*, *Z'´*	2, 1	
ρ_calc_ (g cm^–3^)	1.42240(6)	
T (K)	298	
data collection		
diffractometer	STADI P	
monochromator	Ge(111)	
wavelength (Å)	1.54056	
2θ range (°)	3.000–80.735	
step size (°)	1.05	
time per step (s)	300	
refinement		
number of data points	5183	
number of contributing reflections	567	
*R*_Bragg_ (%)	1.867	
*R*_wp_ (%)	3.510	
χ^2^	2.007	

The crystal structure of MEH (Figure 3) consists of one formula unit in the asymmetric unit (*Z*′ = 1) and two formula units in the unit cell (*Z* = 2). The crystal structure was validated using the Mogul geometry check, available in the “CSD-Core” module within Mercury (version 2024.2.0^36^). An assessment of bond lengths, angles, ring geometries, and torsion angles in the Crystal Structure Database (CSD) revealed a good statistical distribution of similar fragments and confirmed the 3D geometries. The geometrical analysis was also validated by PLATON,^44^ indicating that the terminal NH_3_ (N6) was protonated, interacting with the deprotonated chloride (Cl3).

**Figure 3:**
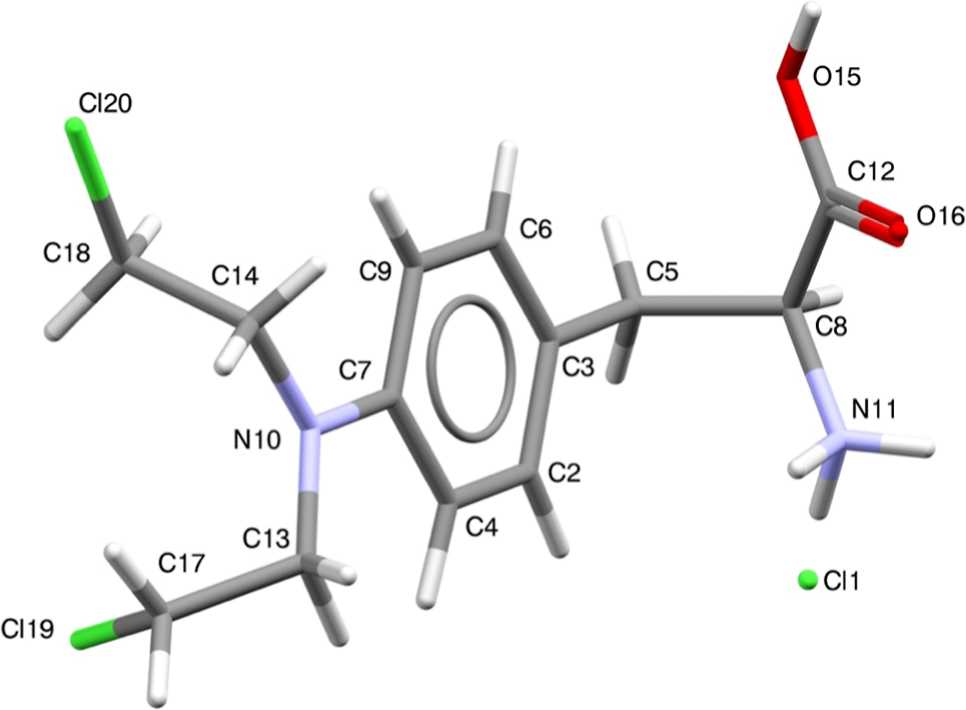
Crystal structure of MEH displaying atom numbering for all non-hydrogen atoms.

### 3.2 Hirshfeld Surface Analysis

Hirshfeld surface analysis (Figure 4) was performed using CrystalExplorer21.^45^ The surfaces were obtained using a standard (high) surface resolution with three-dimensional *d*_*norm*_ surfaces mapped over a fixed color scale of −0.5485 (red) to 1.4093 (blue). The structure revealed at least four visible (out of six hydrogen-bond interactions, as shown in Table S4) red spots, indicating strong interactions. The most relevant (∼3.0 Å) involves atoms O15–H19···Cl1 (left-bottom in Figure 4).

**Figure 4:**
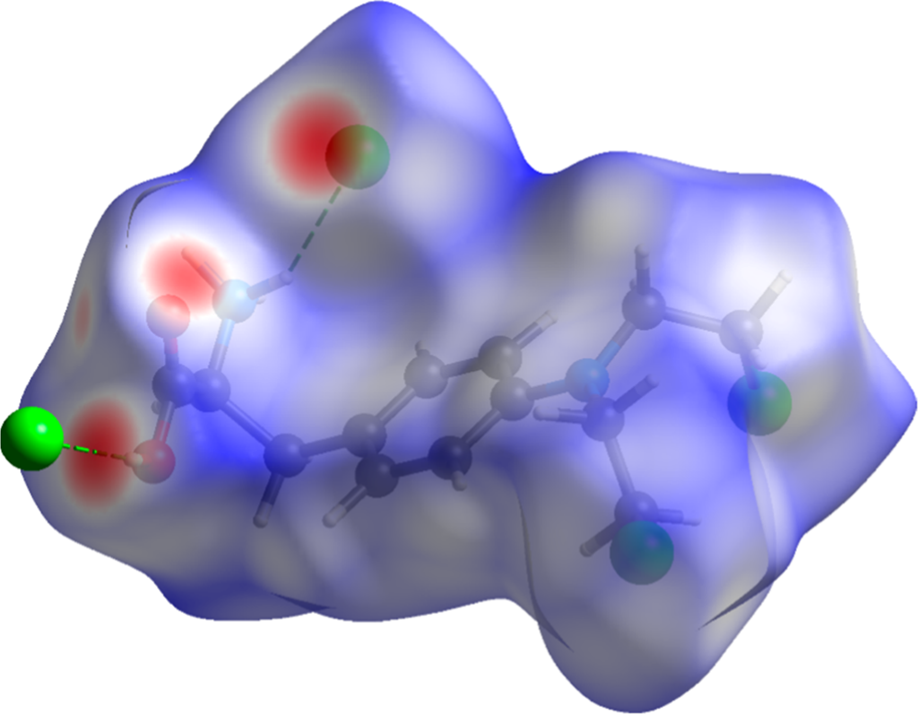
Three-dimensional Hirshfeld surface analysis of MEH plotted over *d*_norm_ from −0.5485 (red) to 1.4093 (blue).

The two-dimensional fingerprint plots (in *d*_e_ vs *d*_i_ coordinates) (Figure 5) show that the contributions to the packing were H···Cl (40.9%), H···H (31.8%), H···O (11.8%), H···C (10.9%), Cl···Cl (2.4%), O···Cl (1.4%), C···Cl (0.3%), O···O (0.2%), H···N (0.2%), N···C (0.2%), and C···C (0.1%).

**Figure 5:**
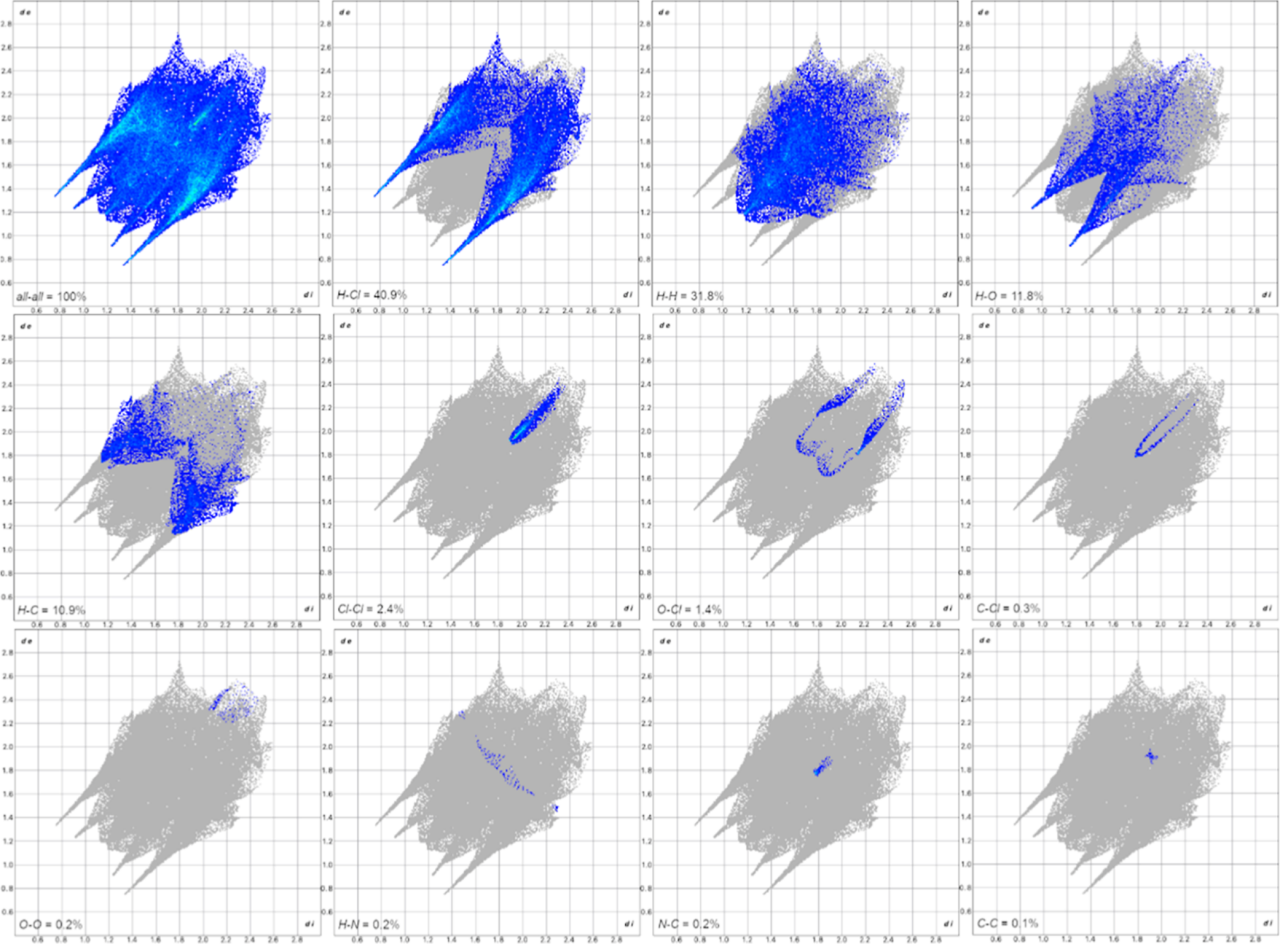
Complete two-dimensional Hirshfeld surface analysis of MEH displaying the relative contributions of individual contacts.

### 3.3 Quantitative Phase Analysis

The powder X-ray diffraction patterns of the samples obtained using the ball mill are displayed in Figure 6. Despite the superposition of the patterns in Figure 6A, we can observe the characteristic peaks of each of the MEH (black line) and CAF (red line) samples in Figure 6B. The most intense peak of MEH in this enlarged region can be seen at ∼13° (2θ). It can also be seen in the diffractograms of the mixtures of MEH and CAF, but with broader peak shapes. This can be attributed to the reduction of crystallite sizes and microstrain effects generated during the ball mill process.

**Figure 6:**
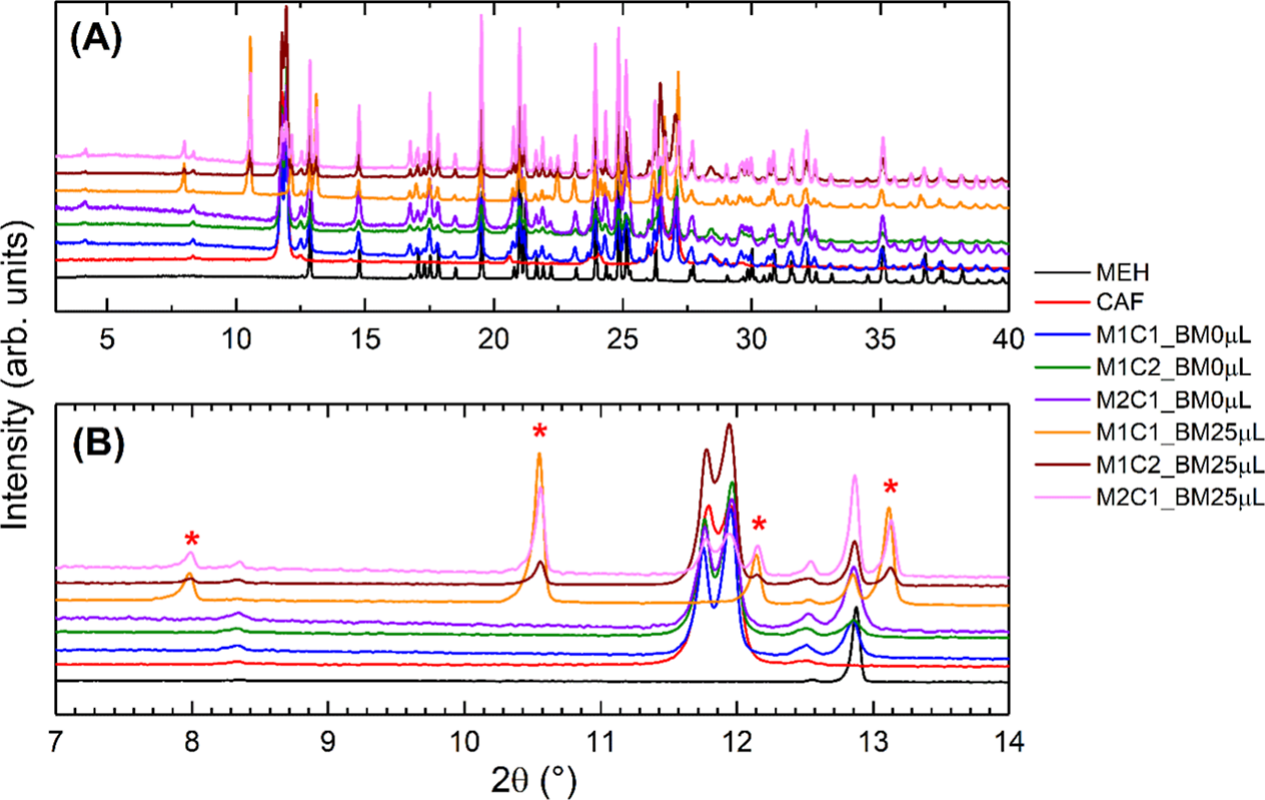
(A) Powder X-ray diffraction patterns of the MEH (black line), CAF (red line), and mixtures of MEH/CAF, with different molar ratios, using the ball mill (BM) without (0 μL) and with (25 μL) the addition of water. (B) Enlarged region of the X-ray diffraction patterns displaying some of the characteristic peaks of MEH and CAF and the formation of a hydrated form of caffeine (indicated by *).

On the other hand, the most intense peaks of CAF can be observed at ∼11.5–12° (2θ). Following the same observation, they are also present in all samples except the M1C1_BM25 μL. In samples M1C2_BM25 μL and M2C1_BM25 μL, we can also see some additional peaks at ∼8 and ∼10.5° (2θ). We could identify them as the formation of a hydrated form of caffeine. Most interesting is the complete formation of such a phase in the M1C1_BM25 μL sample. Quantitative phase analyses using the Rietveld method^46,47^ of the mixture samples in Table 1 are displayed in the Supporting Information material (Figures S1–S12).

We could not observe the formation of the hydrated form of caffeine in the samples prepared using the agate mortar and pestle. For this reason, we do not show the powder X-ray patterns stacked as we did for the samples obtained in the ball mill. This effect is probably related to the higher mechanical energy employed in the ball mill than in manual grinding. The presence of a hydrated caffeine form impacts the biological in vitro assays (discussed below).

### 3.4 FTIR

Figure 7 shows the FTIR spectra of the MEH and CAF samples and the physical mixture between them in a molar ratio of 1:1, carried out in a ball mill without adding water (M1C1_BM0 μL). Tables S5–S7 in the Supporting Information material contain the assignment of the most significant bands for the produced samples. We can observe that the MEH and CAF bands are unchanged in the sample containing their mixtures, which leads us to infer that there was no formation of a new crystalline form. In Figures 8 and 9A,B, it is possible to confirm the presence of the caffeine molecule in the mixture. Bands are observed at 1699 cm^–1^, associated with stretching (ν) of the C=O, C=N, or C=C bonds; CH_3_ (νCH_3_); 1643 cm^–1^ referring to stretching νCH_3_; 1543 cm^–1^ indicative of the stretching vibration νC=N; 1028 cm^–1^ attributed to the νC–C stretching vibration and, finally, a band at 484 cm^–1^ referring to the stretching of the C–N bond.^48^ By analyzing the bands above, we can confirm the physical mixture of caffeine with the melphalan hydrochloride molecule; however, their overlap leads us to believe that there was no formation of a new phase, corroborating the data obtained by X-ray diffraction.

**Figure 7:**
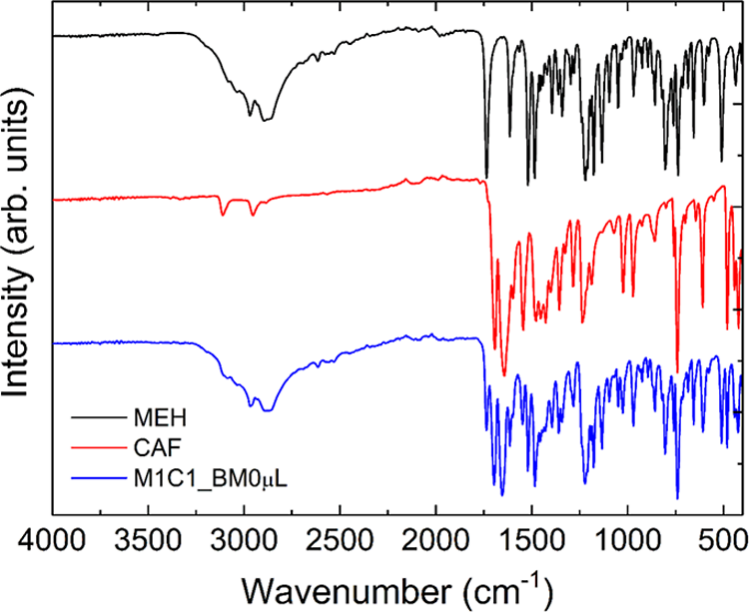
FTIR spectra of MEH (black line), CAF (red line), and M1C1_BM0 μL.

**Figure 8:**
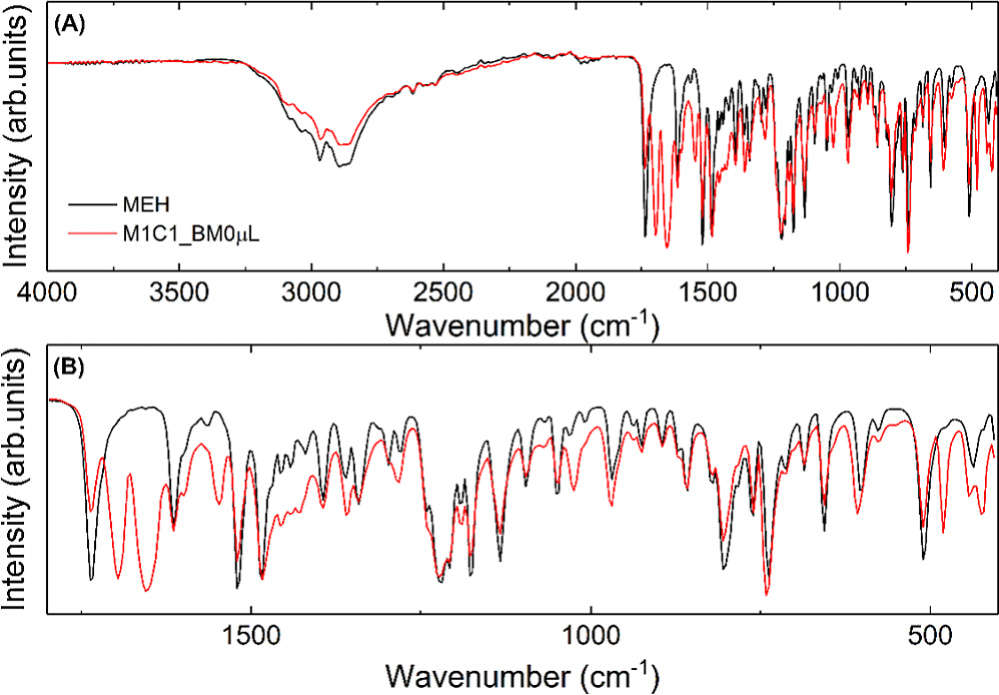
(A) Overlay of the spectra of MEH (black line) and M1C1_BM0 μL (red line) samples. (B) Enlarged region with the main bands.

**Figure 9:**
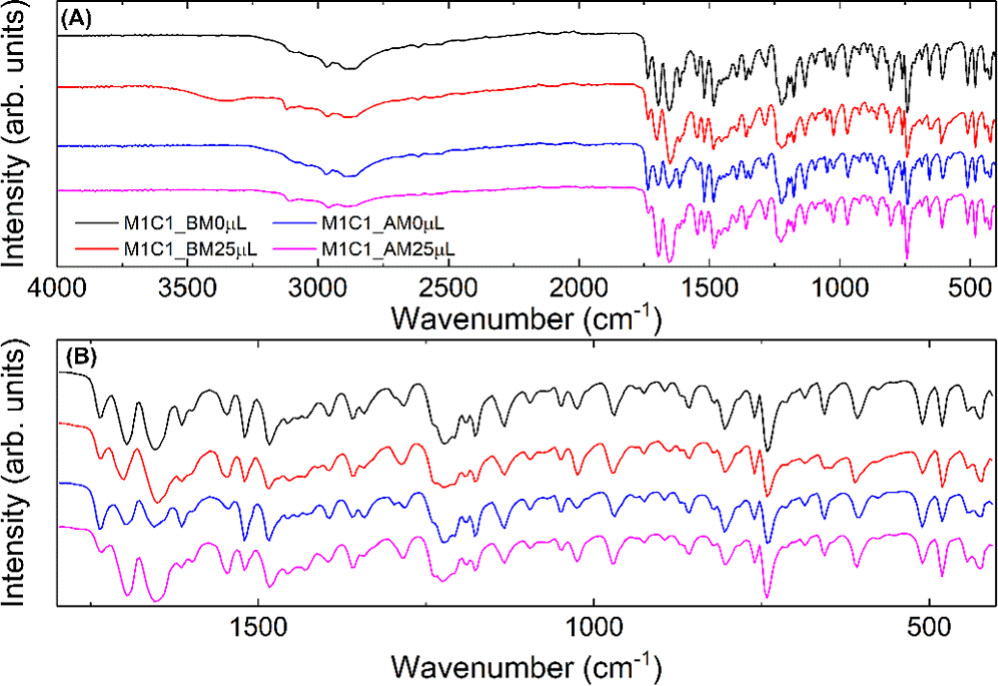
(A) FTIR spectra for M1C1_BM0 μL (black line), M1C1_BM25 μL (red line), M1C1_AM0 μL (blue line), M1C1_AM25 μL (magenta line). (B) Enlarged region with the main bands.

Figure 10 indicates no significant changes in the spectra relating to the samples. Note a slight broadening in the band at 1643 cm^–1^ referring to the CH_3_ stretching vibration of caffeine for the sample containing 25 μL of water and obtained in a ball mill (M1C1_M25 μL). This indicates the formation of hydrated caffeine in the mixture, as observed for all samples obtained in the ball mill with water addition.

**Figure 10:**
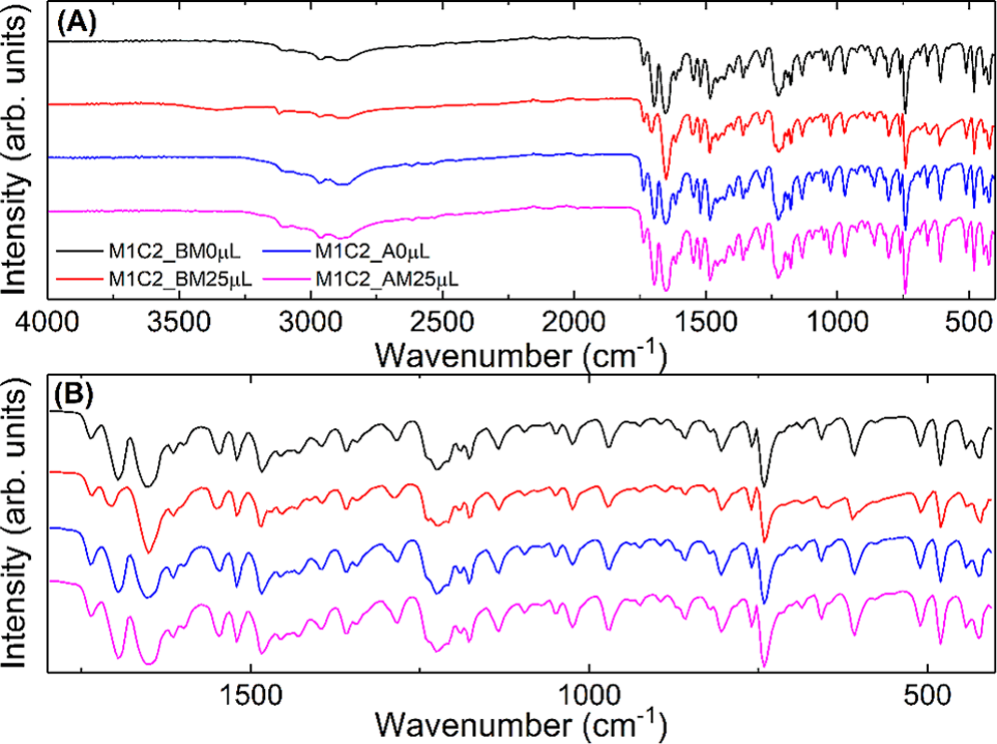
(A) FTIR spectra for MEH and CAF samples in a 1:2 ratio synthesized with a ball mill (BM) and an agate mortar (AM): M1C2_BM0 μL (black line), M1C2_BM25 μL (red line), M1C2_AM0 μL (blue line), and M1C2_AM25 μL (magenta line). (B) Enlarged region with the main bands.

The same behavior can still be observed in the 1643 cm^–1^ band for samples with different caffeine concentrations, as shown in the spectra in Figure 10.

Figure 11 shows a difference in the bands at 1731 cm^–1^ (ν_sym_ C=O) and 1709 cm^–1^ (ν_sym_ C=O), referring to the melphalan hydrochloride molecule. This fact is justified since the concentration of this molecule increased for this set of samples. The change in bands is more evident for the M2C1_M25 μL sample. Therefore, a qualitative analysis of the FTIR spectra suggests only a physical interaction between the molecules.

**Figure 11:**
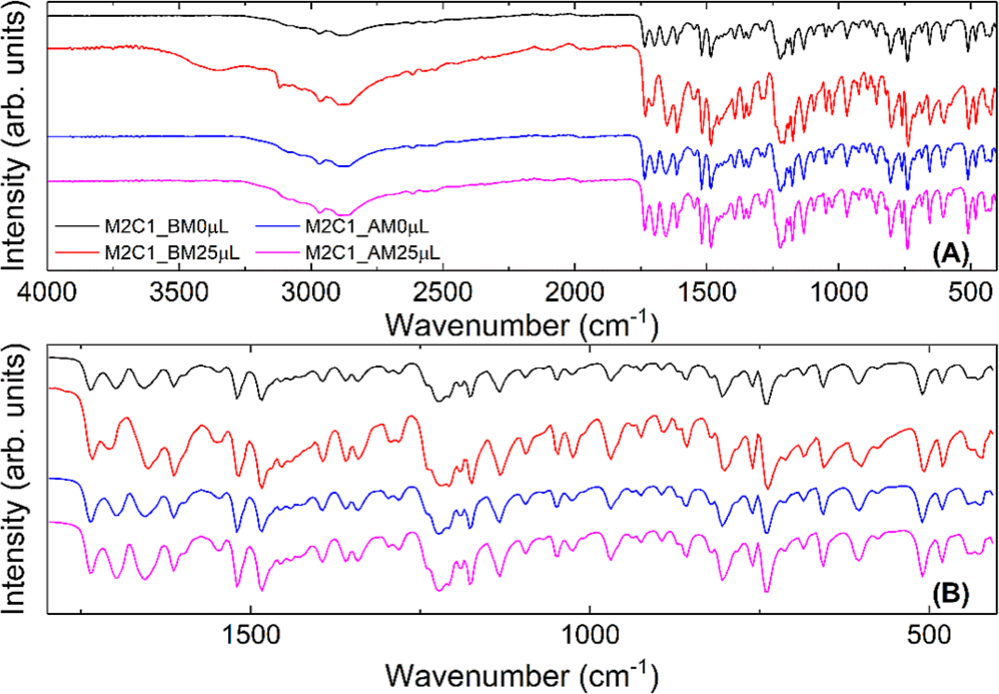
(a) FTIR spectra for MEH and CAF samples in a 2:1 ratio synthesized with a ball mill (BM) and an agate mortar (AM): M2C1_BM0 μL (black line), M2C1_BM25 μL (red line), M2C1_AM0 μL (blue line), and M2C1_AM25 μL (magenta line). (B) Enlarged region with the main bands.

### 3.5 Molecular Dynamics

Given that CAF and MEH could mainly interact via their aromatic groups (imidazole and benzene, respectively), the distance between these two chemical scaffolds was measured throughout the MD, setting a cutoff value of 4 Å, which has been reported for π-stacking interactions.^49^

Figure 12 shows the distance between CAF and MEH through the MD. The red dotted line indicates the cutoff distance. From this analysis, 23 MEH-CAF binding poses (Figure 13) can be extracted from the simulation. Both compounds interact mainly via aromatic groups, although some poses also show interactions between aromatic rings and aliphatic chains. Figure S13 shows a UV–vis study describing the possible interaction of MEH and CAF.

**Figure 12:**
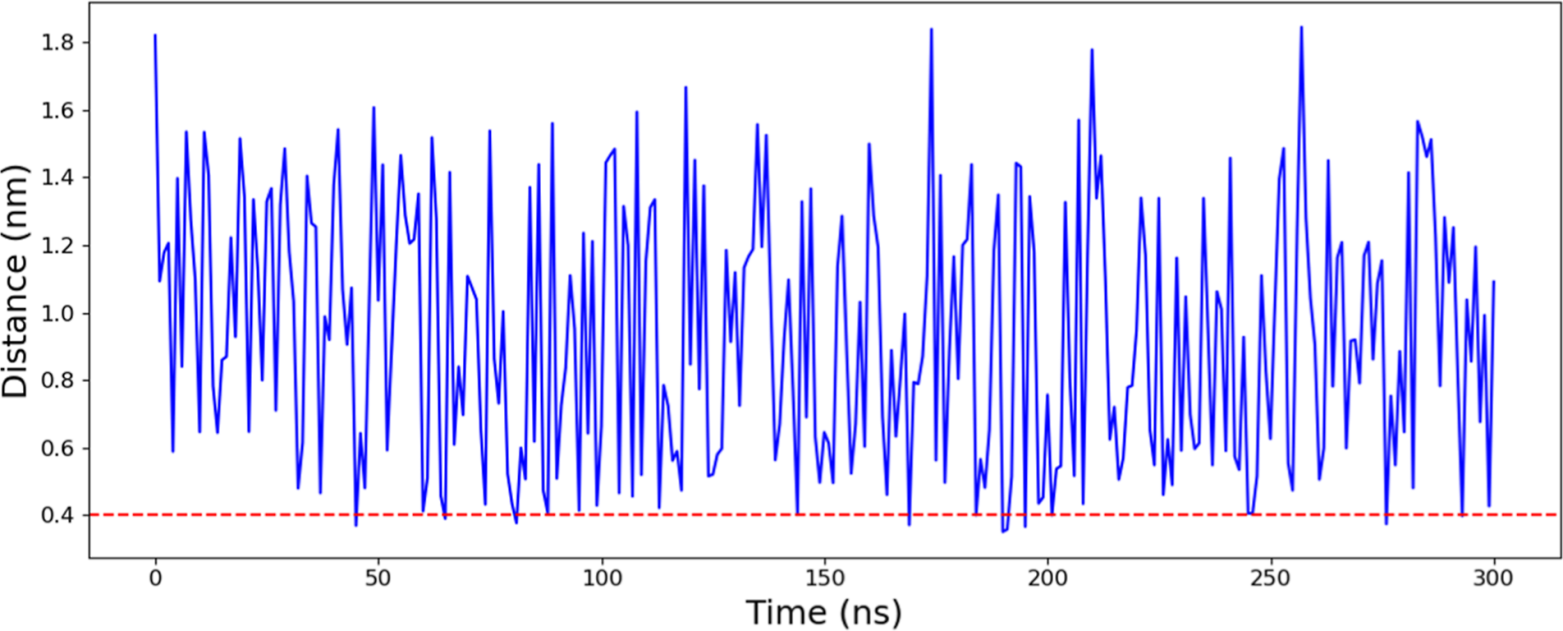
MEH-CAF distances in the MD simulation. The red dotted line shows the cutoff value for the extracted binding poses.

**Figure 13:**
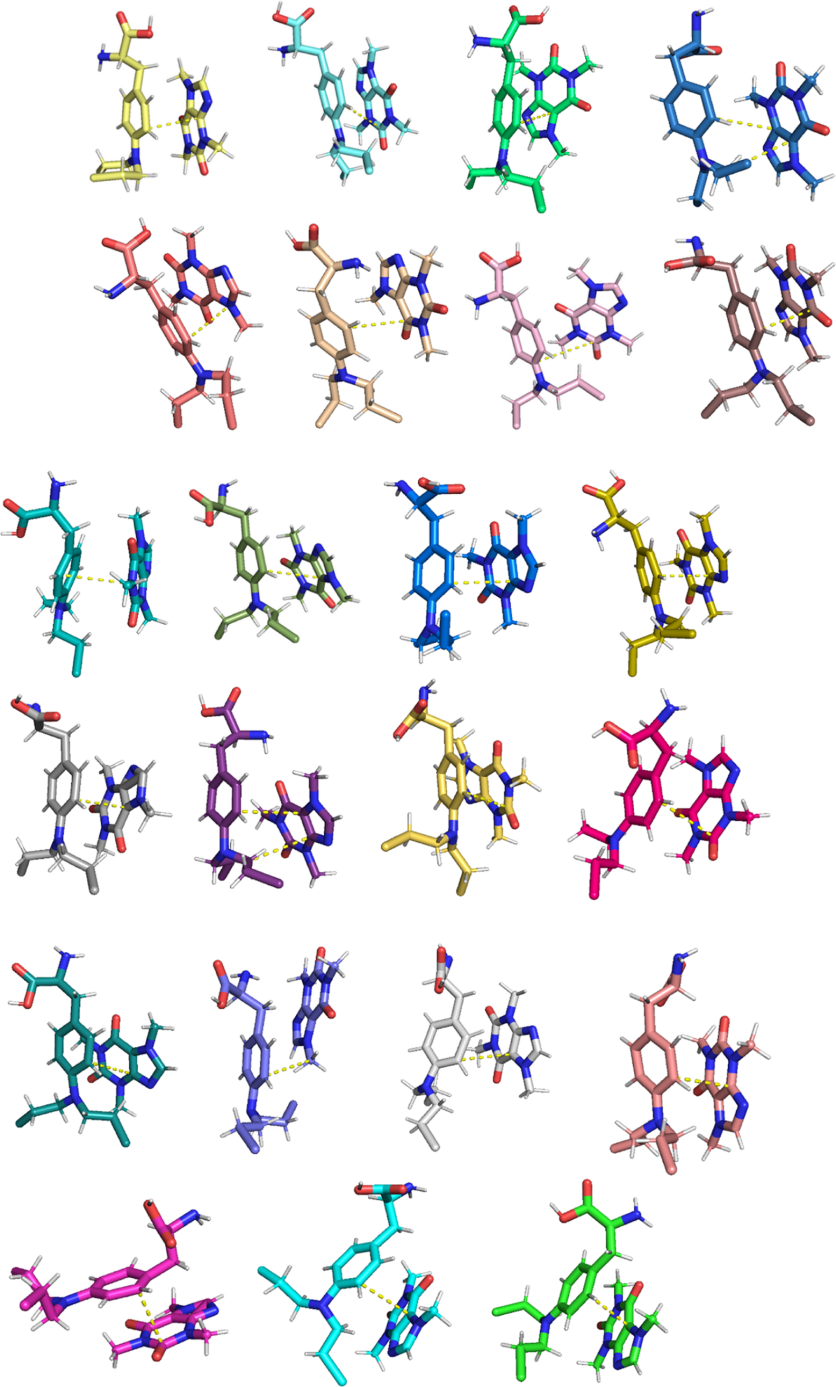
Representative MEH-CAF interacting conformations extracted from MD.

### 3.6 Cell Viability Assessment

Widely used in the literature,^40^ the evaluation of cell viability through the MTT assay aimed to verify the interaction of samples with healthy MRC-5 cells in comparison with HeLa tumor cells for a 24 h incubation period at the same concentrations (2.00, 1.00, 0.50, 0.10 and 0.01 mg mL^–1^). To perform the analysis, the samples were prepared in advance due to the low solubility of MEH, diluted in DMEM culture medium without FBS supplementation, and placed in ultrasound for 1:30 h, and immediately after; they were agitated “overnight.”

Three controls were used as distinct reference points for evaluating the cytotoxic effect. The “control” column refers to cells without any interaction, while the “MEH” and “CAF” columns refer to the respective reagents in the same concentrations as the samples studied. The graphs in Figure 14 show the samples’ interaction with healthy MRC-5 cells.

**Figure 14:**
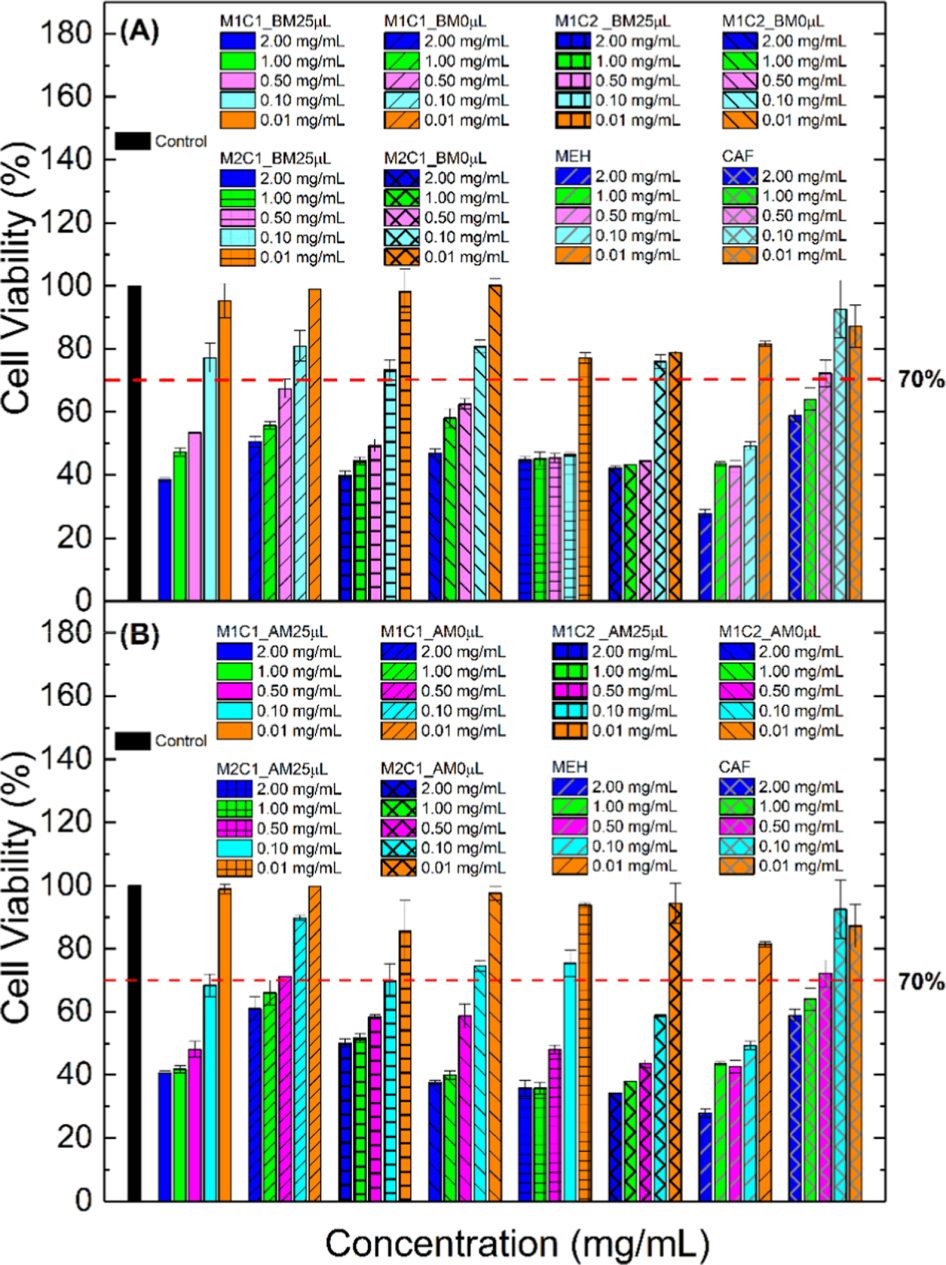
Cell viability in MRC-5 strains of samples synthesized (A) in the ball mill and (B) in a mortar.

Initially, the investigation was carried out to compare the interaction of the MEH/CAF sample with healthy MRC-5 cells, and we can observe that CAF presents toxicity at the highest concentrations (2.00 and 1.00 mg mL^–1^). In comparison, MEH presents viability only at the concentration of 0.01 mg mL^–1^ (Figure 14a). For this cell line, the result shows that the samples synthesized in the presence of water tend to be more toxic when compared to the samples in the absence of water, something that we believe is linked to the effect of osmosis^49^ since the water may be facilitating the entry of the samples into the cells and, consequently, increasing their toxicity. Both the sample synthesized in the ball mill and the one synthesized in the mortar at the concentration of 0.50 mg mL^–1^ presented viability very close to the minimum acceptable (70%); this result is exciting since the concentration studied is very close to that indicated in the package insert for MEH (0.10–0.60 mg kg^–1^ of weight).

In a second step, the investigation was carried out to compare the interaction of the MEH/CAF sample with HeLa tumor cells at the same concentrations. Figure 15 shows that CAF is viable at all concentrations analyzed, while MEH is only nonviable at the highest concentrations (2.00 and 1.00 mg mL^–1^). We observed that both the samples synthesized in the ball mill and those synthesized in a mortar in the presence and absence of water present greater viability at the lowest concentrations (0.10 and 0.01 mg mL^–1^) regardless of the molar fraction of the reagents. At the highest concentrations (2.00 and 1.00 mg mL^–1^), they present toxicity in all samples analyzed.

**Figure 15:**
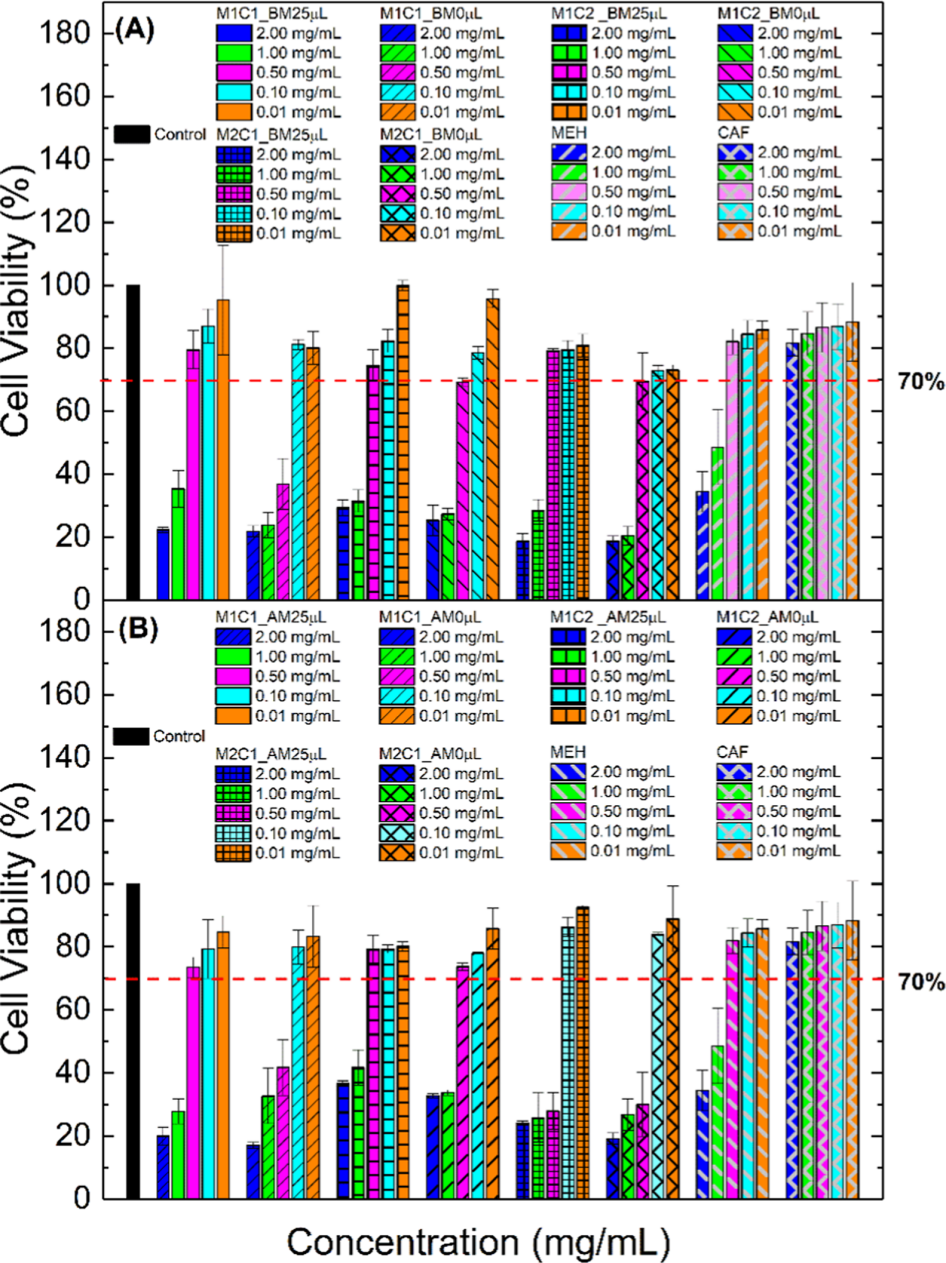
Cell viability in the HeLa cell line of samples synthesized (A) in the ball mill and (B) in a mortar.

We have a peculiarity in the samples of 0.50 mg mL^–1^ since the concentration presented the most variable result. In the samples synthesized in the ball mill, only M1C1_BM0 μL showed toxicity (∼40%; Figure 15a), while the other samples were at the minimum acceptable limit of 70%. Concerning the samples synthesized in the mortar, M1C1_AM0 μL showed a similar result to those synthesized in the ball mill. However, in the M2C1 samples, in the presence and absence of water, the concentration of 0.50 mg mL^–1^ showed toxicity (∼35%).

When we compare the study in the two cell lines, we understand that the intention is to have the lowest concentration of MEH to avoid adverse effects but maintain the ability to eliminate tumor cells. Therefore, from the analyses performed so far in the HeLa cells, the samples M1C1_25 μL, M1C2_25 μL, and M1C2_0 μL stand out for being within the permitted limit (70% viability) regardless of whether the synthesis is performed using a mortar or ball mill, as shown in Figure 16.

**Figure 16:**
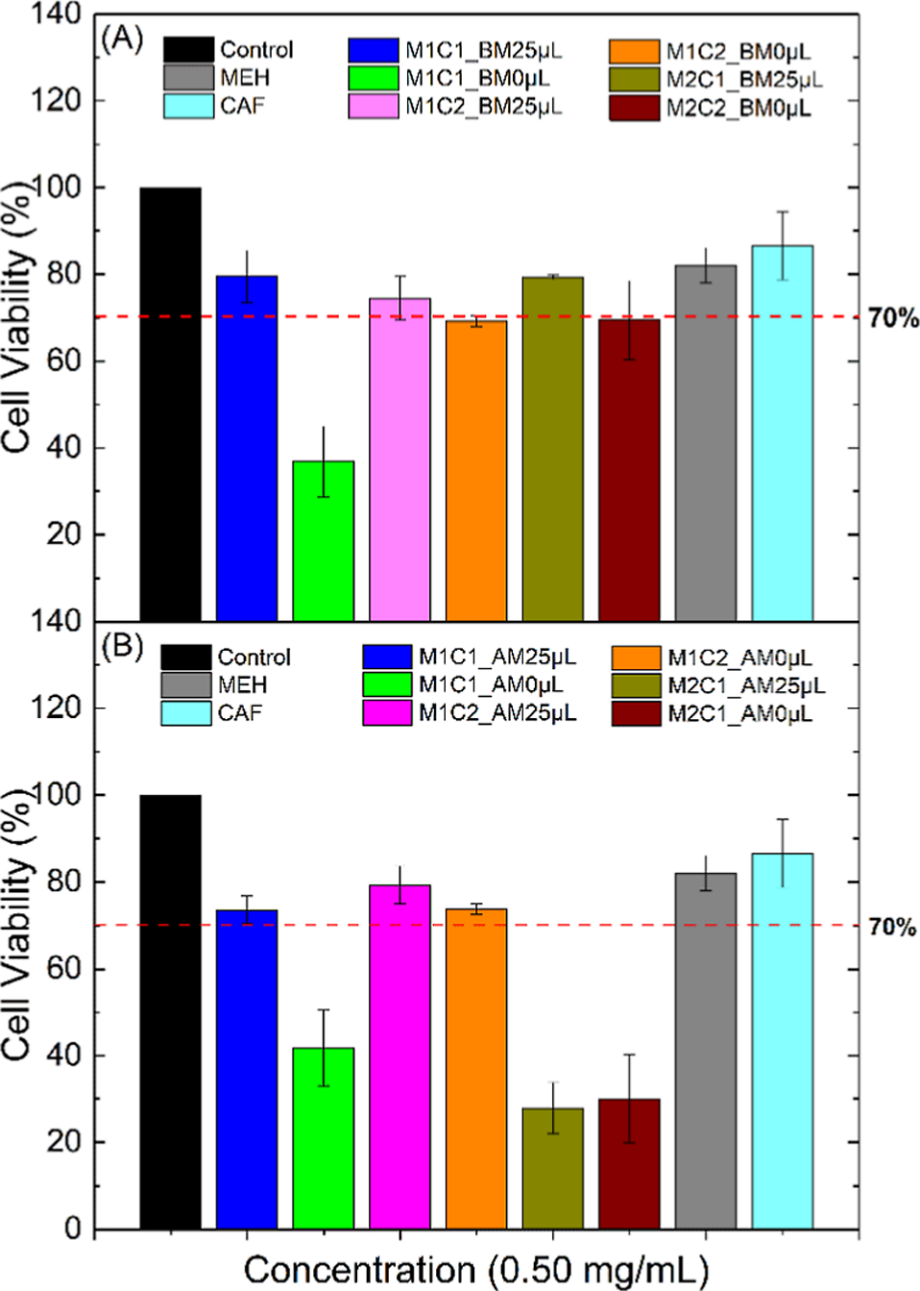
Cell viability results in the HeLa cell line at a concentration of 0.50 mg mL^–1^ synthesized in (A) the ball mill (BM) and (B) the agate mortar (AM).

Figure 17 shows that only the sample M1C1_0 μL showed viability for both syntheses; however, the synthesis in the ball mill was at the limit (considering the standard deviation). This result was already expected since the MRC-5 cells are healthy and do not have high multiplication, which makes them more susceptible to MEH/CAF.

**Figure 17:**
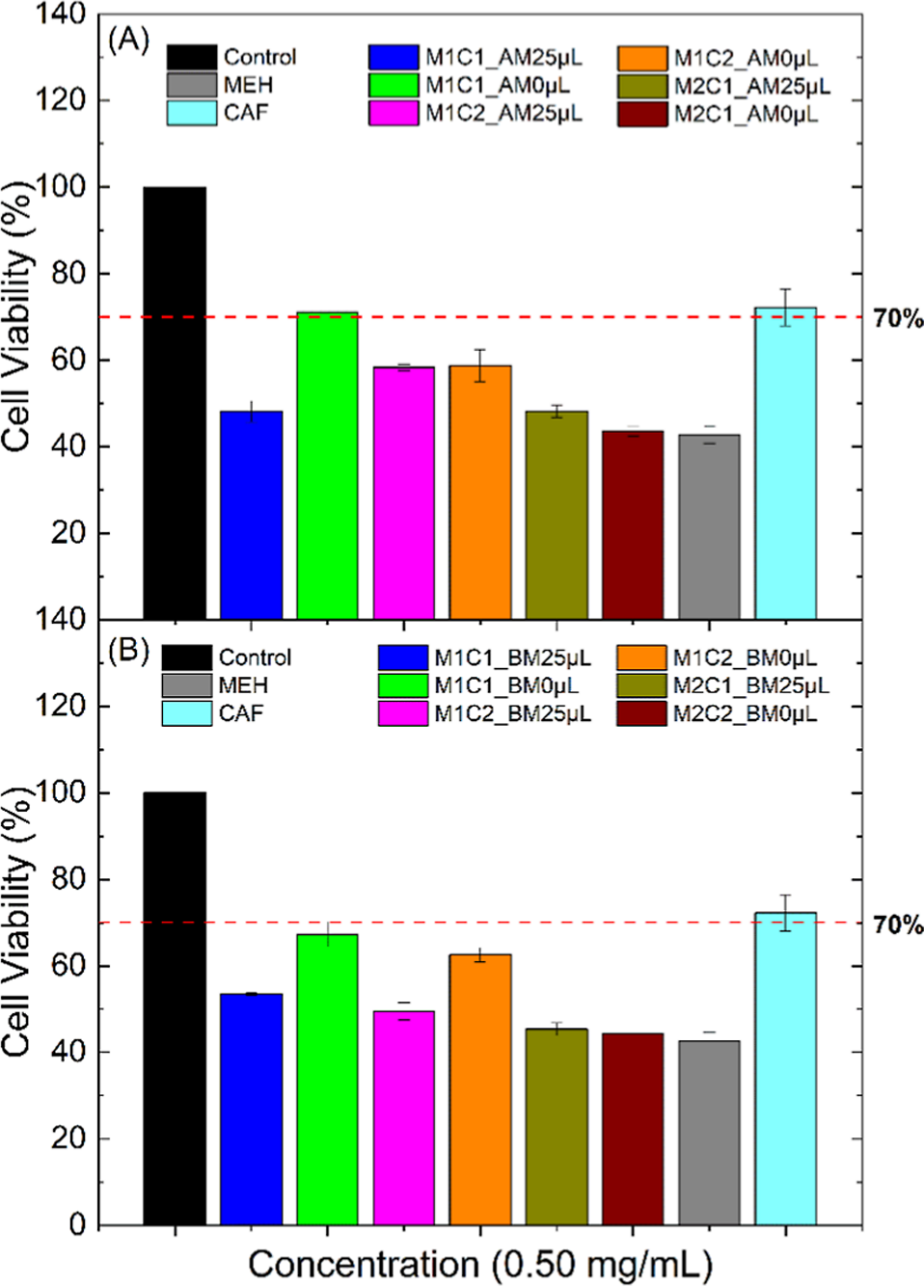
Cell viability results in the MRC-5 cell line at a concentration of 0.50 mg mL^–1^ synthesized in (A) the ball mill (BM) and (B) the agate mortar (AM).

In this context, based on the viability graphs, when we compare the study in the two cell lines, we understand that the intention is to have the lowest concentration of MEH to avoid adverse effects and maintain the ability to eliminate tumor cells. Therefore, from the analyses performed so far, the synthesis by ball mill of the M1C1_BM0 μL sample would be the most interesting.

## 4 Conclusion

We successfully determined the crystal structure of melphalan hydrochloride using powder X-ray diffraction data and a simulated annealing approach. The final structure was refined with the Rietveld method, which revealed adequate statistical parameters and a good visual fit to the data. Molecular dynamics simulations indicate that the association of MEH and CAF primarily occurs through aromatic groups. During synthesis, we combined melphalan hydrochloride with caffeine using a ball mill and an agate mortar, both in the presence and absence of water. The samples were tested in a biological environment to evaluate cell viability across different cell lines and concentrations. Although we could not obtain any cocrystals between MEH and CAF, their interaction favors a cytotoxic effect in rapidly dividing cells, such as cancer cells, while showing good viability in healthy cells. These results highlight the potential of supramolecular interactions as a promising strategy to enhance the therapeutic efficacy of poorly water-soluble drugs, opening new perspectives for developing more efficient and targeted treatments against cancer. This study paves the way for future studies to obtain potential cocrystals.

## Supplementary Material



## Notes

Supporting Information contains tables displaying atomic coordinates, bond lengths, valence angles, hydrogen-bonding information, FTIR tables assigning the most significant bands in the spectra, Rietveld plots of all produced samples, and UV–vis spectra (PDF)
