# Supplementary material for: Crystal Structure of Melphalan Hydrochloride and Its Association with Caffeine Improves Its Antineoplastic Action
Source: ACS Omega. 2025 May 15;10(20):20661–73. doi: 10.1021/acsomega.5c01538 (PMC12120582; doi:10.1021/acsomega.5c01538)
Supplement: Supplementary file 1 [file ao5c01538_si_001.pdf]

Crystal structure of melphalan hydrochloride and its association with caffeine improves its antineoplastic action

Juliana Pereira da Silva[1], Carin Cristina da Silva Batista[1] , Maria Lúcia Schumacher[2], Santiago Rodriguez[3], Alan Talevi[3], Paula Haddad[2], Guillermo Raul Castro[1,3], and Fabio Furlan Ferreira[1,3*]

[1]Centro de Ciências Naturais e Humanas (CCNH), Universidade Federal do ABC (UFABC), Av. dos Estados, 5001, Santo André, SP, 09280-560, Brazil
[2]Instituto de Ciências Ambientais, Químicas e Farmacêuticas, Departamento de Química, Universidade Federal de São Paulo, Rua São Nicolau, 210, Diadema, SP, 09913-030, Brazil
[3]Laboratorio de Investigación y Desarrollo de Bioactivos (LIDeB), Departamento de Ciencias Biológicas, Facultad de Ciencias Exactas, Universidad Nacional de La Plata (UNLP), Calle 47 y 115 (B1900AD), La Plata, Buenos Aires, Argentina
[43]Núcleo de Nanomedicina (NANOMED), Universidade Federal do ABC (UFABC), Av. dos Estados, 5001, Santo André, SP, 09280-560, Brazil

Supporting information

[*] Corresponding author: fabio.furlan@ufabc.edu.br

Table S1. Atomic coordinates for MEH.

Atom	x	y	z	U_{eq} (Å^2)
Cl19	-0.0212(4)	0.5717(5)	0.10192(19)	0.2992
Cl20	0.4984(5)	-0.0522(5)	0.0371(2)	0.2992
Cl1	0.2643(4)	0.2969(5)	0.42729(18)	0.2992
O15	-0.1244(7)	-0.4229(6)	0.3807(3)	0.2992
O16	0.1052(8)	-0.2213(7)	0.4410(3)	0.2992
N10	0.2669(3)	0.2496(4)	0.18151(11)	0.2992
N11	-0.2563(9)	0.0317(7)	0.4430(3)	0.2992
C2	-0.2203(5)	0.1788(6)	0.29976(16)	0.2992
C3	-0.2504(5)	-0.0104(5)	0.29869(14)	0.2992
C4	-0.0491(6)	0.2658(6)	0.26121(15)	0.2992
C5	-0.4298(7)	-0.1045(7)	0.33948(18)	0.2992
C6	-0.1080(6)	-0.1114(6)	0.25892(15)	0.2992
C7	0.0980(3)	0.1606(4)	0.22058(11)	0.2992
C8	-0.3294(9)	-0.1410(6)	0.4092(2)	0.2992
C9	0.0639(6)	-0.0278(6)	0.21997(13)	0.2992
C12	-0.0932(8)	-0.2654(6)	0.4124(3)	0.2992
C13	0.3409(6)	0.4403(5)	0.18975(15)	0.2992
C14	0.4284(6)	0.1484(4)	0.13934(17)	0.2992
C17	0.3172(6)	0.5538(5)	0.1287(2)	0.2992
C18	0.2776(9)	0.0852(9)	0.0786(3)	0.2992
H1	-0.0486(6)	0.4048(6)	0.25975(15)	0.3591
H2	-0.3848(9)	0.1176(7)	0.4435(3)	0.3591
H3	-0.1952(9)	0.0243(7)	0.4822(3)	0.3591
H4	-0.1356(9)	0.1005(7)	0.4229(3)	0.3591
H5	0.1763(6)	-0.0940(6)	0.19416(13)	0.3591
H6	0.4151(6)	0.4983(5)	0.0974(2)	0.3591
H7	0.4076(6)	0.6797(5)	0.1315(2)	0.3591
H8	0.4912(6)	0.0588(4)	0.16295(17)	0.3591
H9	0.5657(6)	0.2308(4)	0.12715(17)	0.3591
H10	0.1875(9)	0.1903(9)	0.0550(3)	0.3591
H11	0.1422(9)	0.0102(9)	0.0917(3)	0.3591
H12	-0.3297(5)	0.2580(6)	0.32478(16)	0.3591
H13	-0.4652(9)	-0.1863(6)	0.4321(2)	0.3591
H14	-0.4863(7)	-0.2132(7)	0.32274(18)	0.3591
H15	-0.5823(7)	-0.0262(7)	0.34124(18)	0.3591
H16	-0.1135(6)	-0.2355(6)	0.25742(15)	0.3591
H17	0.4956(6)	0.4659(5)	0.20597(15)	0.3591
H18	0.2097(6)	0.5149(5)	0.21577(15)	0.3591
H19	0.0185(7)	-0.5013(6)	0.3823(3)	0.3591

Table S2. Bond length data for MEH.

Atom1−Atom2	Length (Å)	Atom1−Atom2	Length (Å)
Cl19−C17	1.817(4)	N11−H3	0.871(9)
Cl20−C18	1.795(7)	N11−H4	0.928(7)
O15−C12	1.330(7)	C13−C17	1.531(5)
O16−C12	1.204(7)	C14−C18	1.531(7)
N10−C7	1.404(3)	C2−H12	0.988(5)
N10−C13	1.444(5)	C4−H1	1.010(6)
N10−C14	1.464(4)	C5−H14	0.906(7)
N11−C8	1.481(7)	C5−H15	0.978(6)
O15−H19	0.935(6)	C6−H16	0.902(6)
C2−C3	1.383(6)	C8−H13	0.942(6)
C2−C4	1.398(5)	C9−H5	0.956(5)
C3−C5	1.480(5)	C13−H17	0.873(4)
C3−C6	1.371(5)	C13−H18	1.055(5)
C4−C7	1.413(4)	C14−H8	0.871(4)
C5−C8	1.556(6)	C14−H9	0.979(4)
C6−C9	1.395(5)	C17−H6	0.952(5)
C7−C9	1.379(5)	C17−H7	1.028(5)
C8−C12	1.522(6)	C18−H10	1.010(9)
N11−H2	0.915(7)	C18−H11	0.946(8)

Table S3. Valence angle data for MEH.

Atom1−Atom2−Atom3	Bond angle (º)	Atom1−Atom2−Atom3	Bond angle (º)
C7−N10−C13	122.8(2)	N10−C14−C18	112.4(3)
C7−N10−C14	122.4(3)	Cl19−C17−C13	108.9(3)
C13−N10−C14	113.3(2)	Cl20−C18−C14	105.6(3)
C12−O15−H19	115.8(5)	C3−C2−H12	121.2(4)
C3−C2−C4	121.0(3)	C4−C2−H12	117.5(5)
C2−C3−C5	121.6(3)	C2−C4−H1	118.2(4)
C2−C3−C6	118.5(3)	C7−C4−H1	121.3(4)
C5−C3−C6	120.0(4)	C3−C5−H14	112.0(4)
C2−C4−C7	120.2(4)	C3−C5−H15	107.4(5)
C3−C5−C8	116.6(3)	C8−C5−H14	107.6(5)
C3−C6−C9	121.6(4)	C8−C5−H15	106.5(4)
N10−C7−C9	122.4(2)	H14−C5−H15	106.2(5)
C4−C7−C9	117.9(3)	C3−C6−H16	122.6(4)
N10−C7−C4	119.7(3)	C9−C6−H16	115.7(4)
N11−C8−C5	111.9(4)	N11−C8−H13	102.9(5)
C5−C8−C12	111.2(4)	C5−C8−H13	109.4(5)
N11−C8−C12	107.5(4)	C12−C8−H13	113.6(5)
C6−C9−C7	120.9(3)	C6−C9−H5	124.0(5)
C8−N11−H2	114.8(6)	C7−C9−H5	114.9(4)
C8−N11−H3	118.3(6)	N10−C13−H17	118.9(4)
C8−N11−H4	113.2(6)	N10−C13−H18	112.3(3)
H2−N11−H3	104.6(8)	C17−C13−H17	103.7(4)
H2−N11−H4	98.9(7)	C17−C13−H18	98.4(3)
H3−N11−H4	104.8(7)	H17−C13−H18	107.2(4)
O15−C12−O16	123.6(4)	N10−C14−H8	103.5(4)
O15−C12−C8	114.6(4)	N10−C14−H9	108.2(3)
O16−C12−C8	121.7(5)	C18−C14−H8	114.2(4)
N10−C13−C17	114.1(3)	C18−C14−H9	107.6(4)
H8−C14−H9	110.8(4)	Cl20−C18−H10	117.9(6)
Cl19−C17−H6	111.4(4)	Cl20−C18−H11	109.7(6)
Cl19−C17−H7	112.6(3)	C14−C18−H10	112.6(6)
C13−C17−H6	110.1(4)	C14−C18−H11	106.0(6)
C13−C17−H7	114.8(4)	H10−C18−H11	104.5(6)
H6−C17−H7	98.8(4)		

Table S4. Hydrogen-bonding information for MEH.

Donor	Acceptor	D-H (Å)	H···A (Å)	D···A (Å)	D−H···A (Å)	Type
**N11−H2**	Cl1	0.915(7)	2.249(6)	3.150(6)	168.2(6)	Intermolecular
**N11−H3**	Cl1	0.871(9)	2.573(7)	3.236(7)	133.6(5)	Intermolecular
**N11−H3**	O16	0.871(9)	2.485(8)	3.100(8)	128.3(6)	Intermolecular
**N11−H4**	Cl1	0.928(7)	2.518(6)	3.357(6)	150.5(6)	intermolecular
**O15−H19**	Cl1	0.935(6)	2.127(6)	2.988(5)	152.6(6)	Intermolecular
**C8−H13**	O16	0.942(6)	2.269(6)	3.115(6)	149.1(5)	Intermolecular

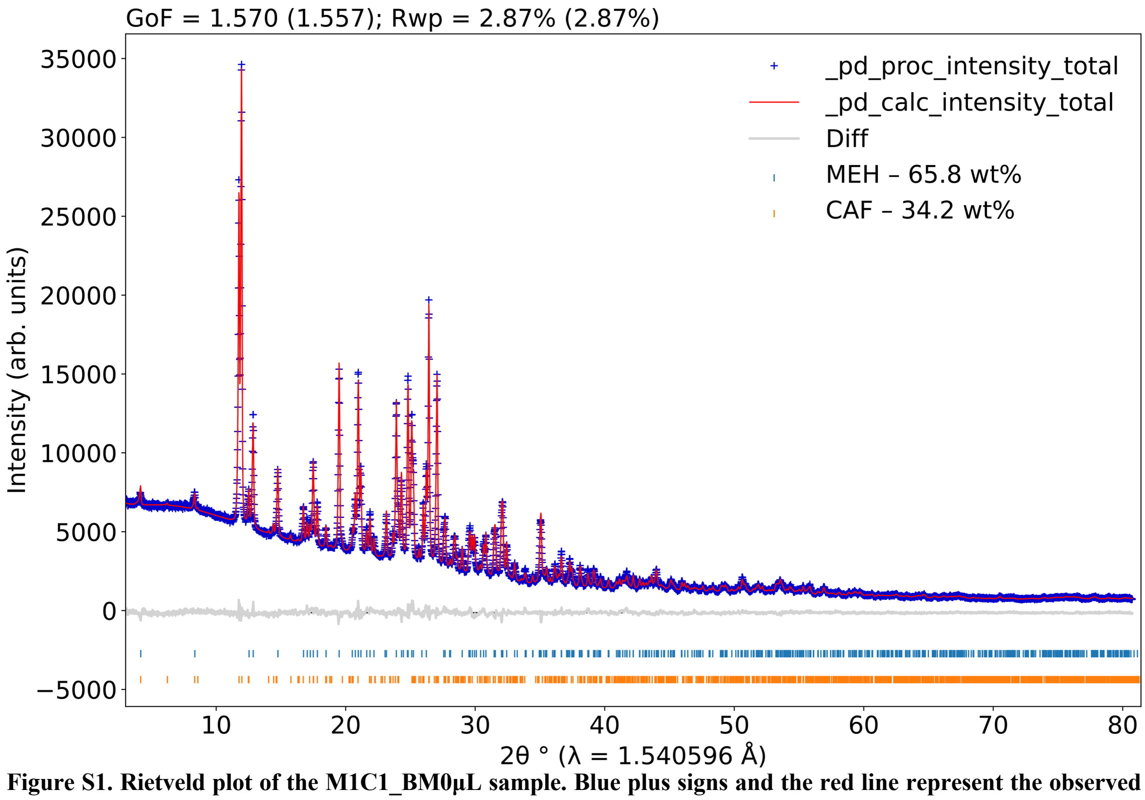

Figure S1. Rietveld plot of the M1C1_BM0µL sample. Blue plus signs and the red line represent the observed and calculated data, respectively. The grey line at the bottom indicates the difference between observed and calculated data. Blue and orange vertical bars represent the melphalan hydrochloride (MEH) and anhydrous caffeine (CAF) phases.

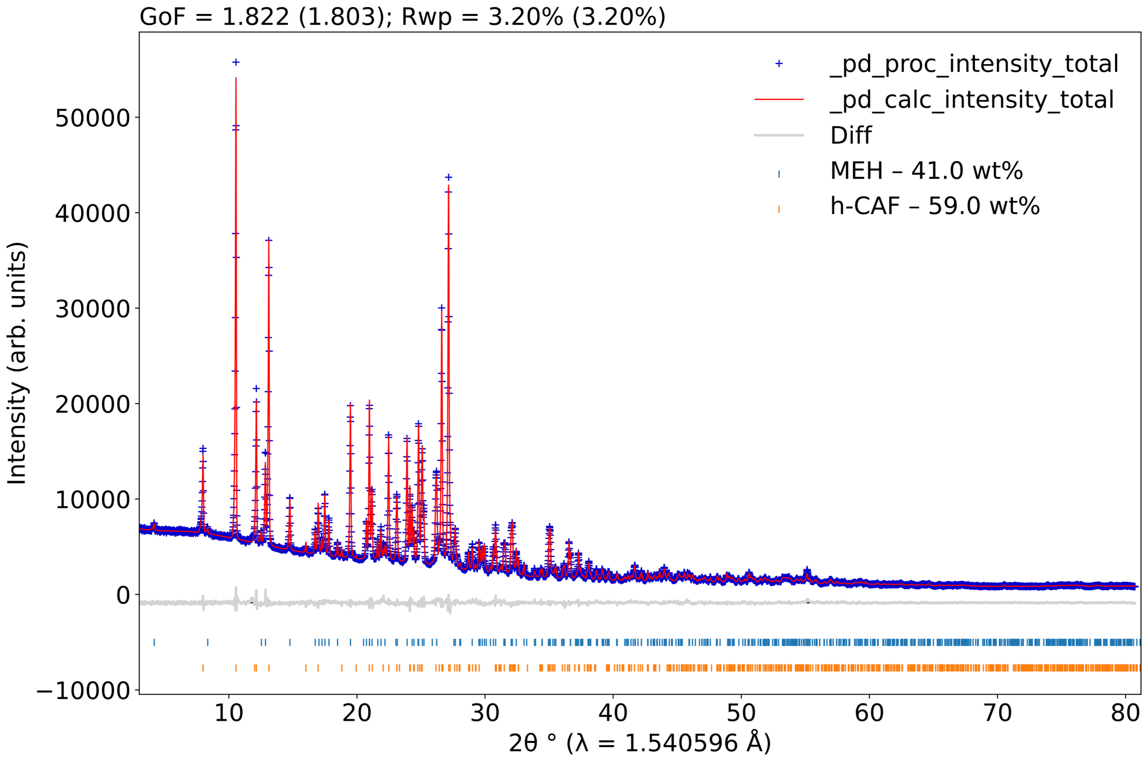

Figure S2. Rietveld plot of the M1C1_BM25µL sample. Blue plus signs and the red line represent the observed and calculated data, respectively. The grey line at the bottom indicates the difference between observed and calculated data. Blue and orange vertical bars represent the melphalan hydrochloride (MEH) and hydrated caffeine (h-CAF) phases.

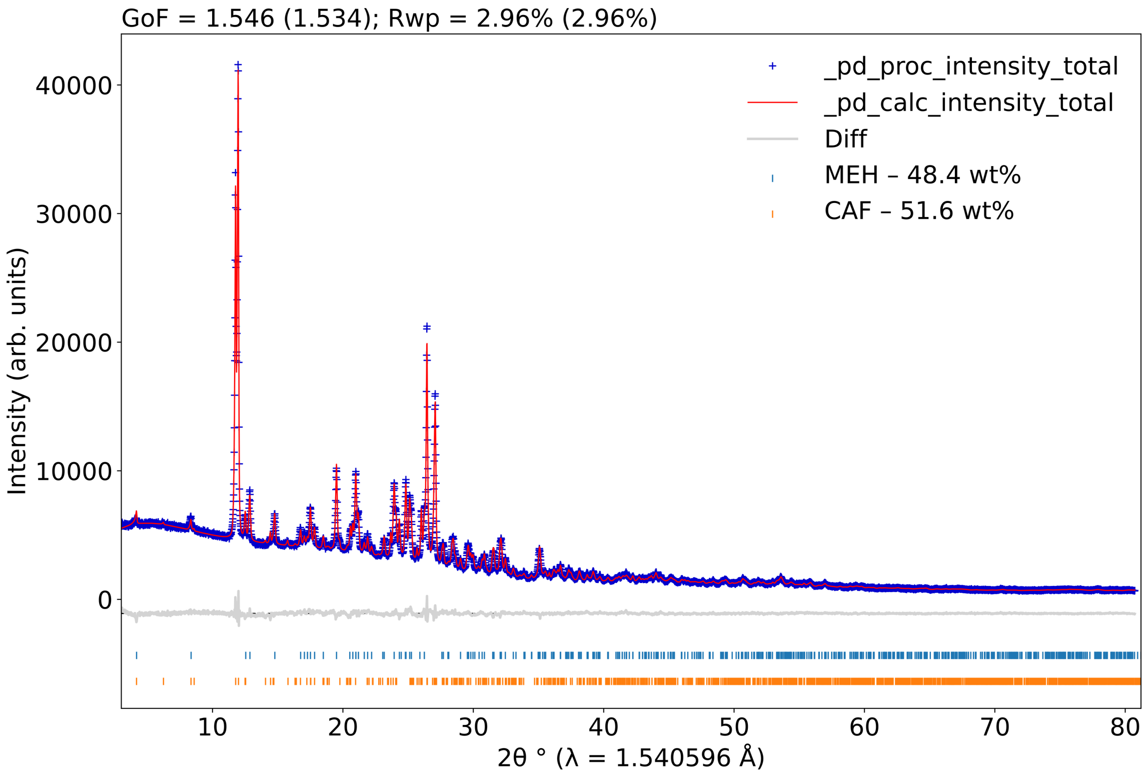

Figure S3. Rietveld plot of the M1C2_BM0µL sample. Blue plus signs and the red line represent the observed and calculated data, respectively. The grey line at the bottom indicates the difference between observed and calculated data. Blue and orange vertical bars represent the melphalan hydrochloride (MEH) and anhydrous caffeine (CAF) phases.

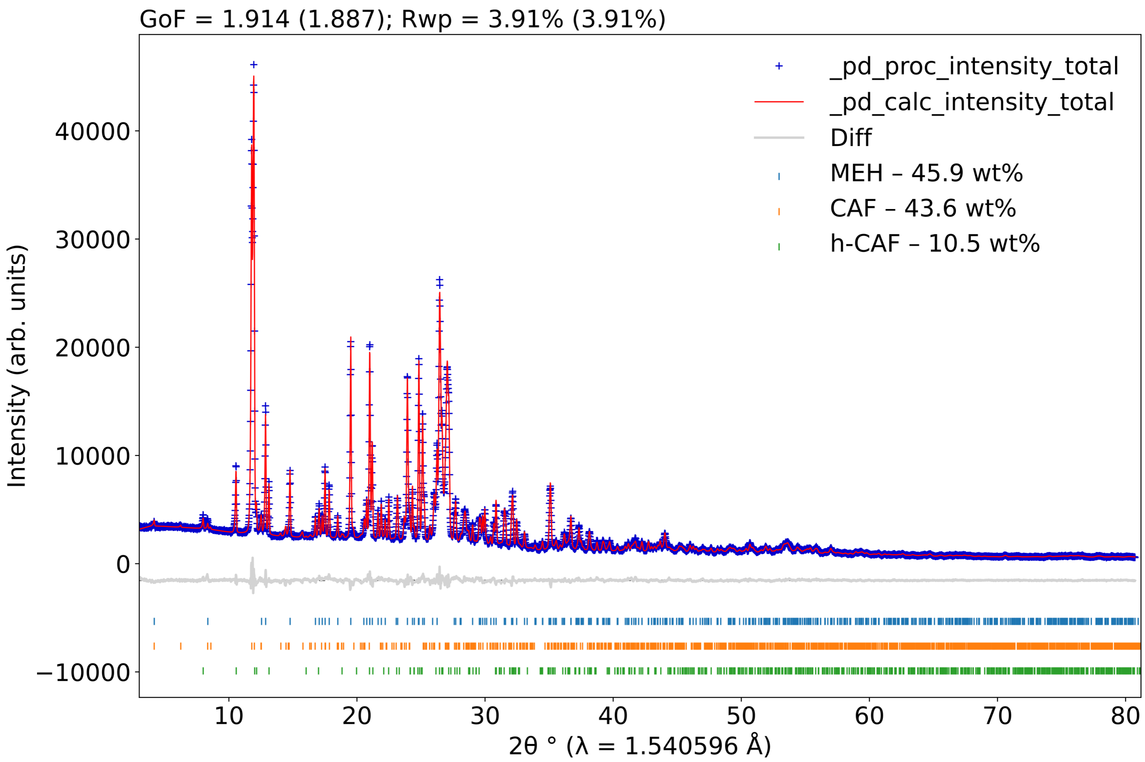

Figure S4. Rietveld plot of the M1C2_BM25µL sample. Blue plus signs and the red line represent the observed and calculated data, respectively. The grey line at the bottom indicates the difference between observed and calculated data. Blue, orange, and green vertical bars represent the melphalan hydrochloride (MEH), anhydrous caffeine (CAF), and hydrated caffeine (h-CAF) phases.

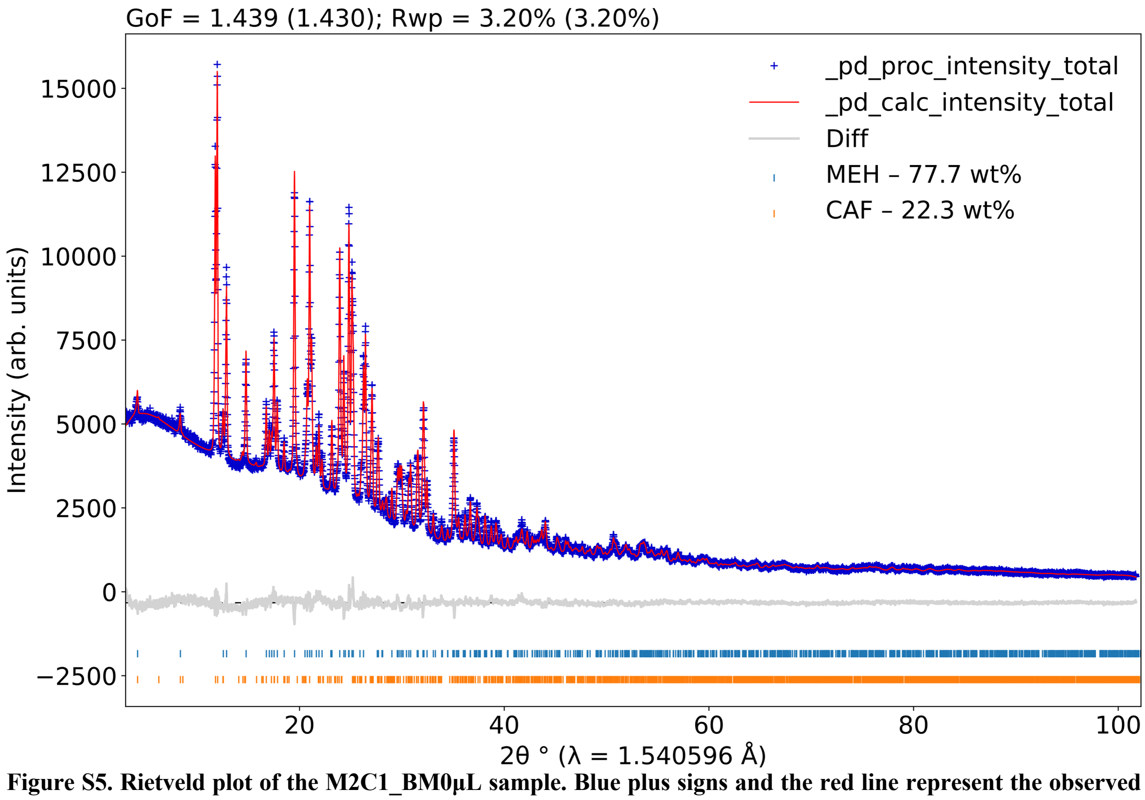

Figure S5. Rietveld plot of the M2C1_BM0µL sample. Blue plus signs and the red line represent the observed and calculated data, respectively. The grey line at the bottom indicates the difference between observed and calculated data. Blue and orange vertical bars represent the melphalan hydrochloride (MEH) and anhydrous caffeine (CAF) phases.

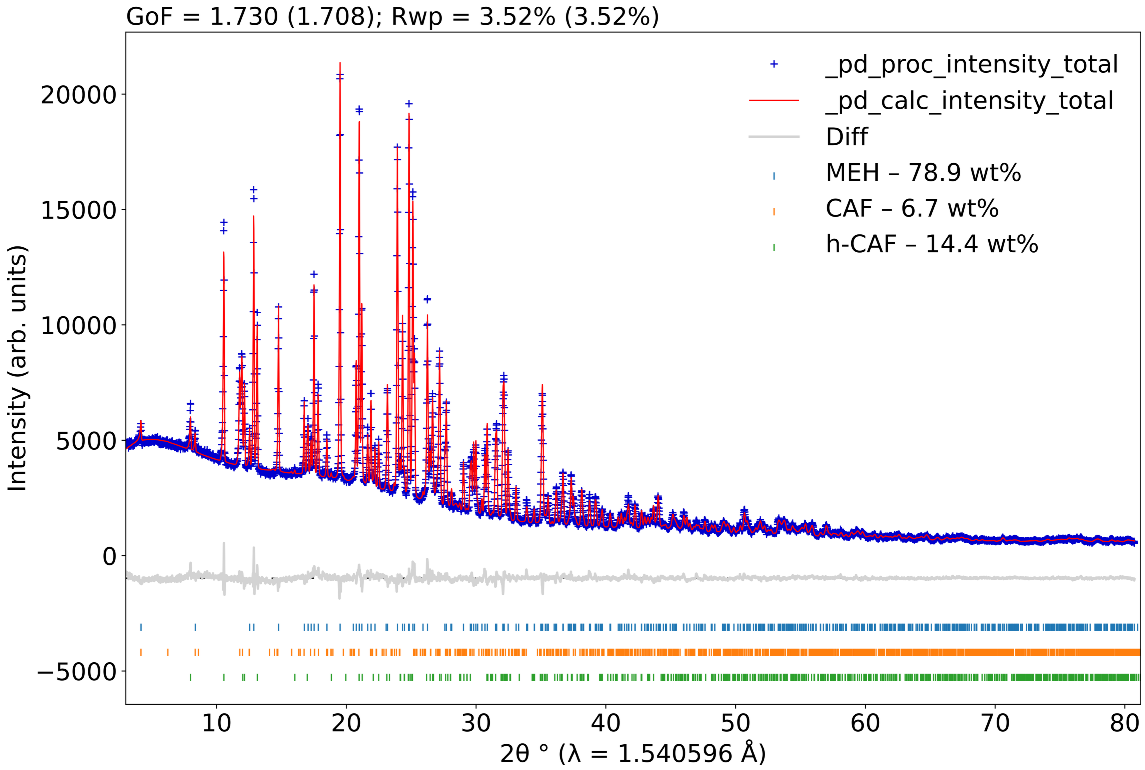

Figure S6. Rietveld plot of the M2C1_BM25µL sample. Blue plus signs and the red line represent the observed and calculated data, respectively. The grey line at the bottom indicates the difference between observed and calculated data. Blue, orange, and green vertical bars represent the melphalan hydrochloride (MEH), anhydrous caffeine (CAF), and hydrated caffeine (h-CAF) phases.

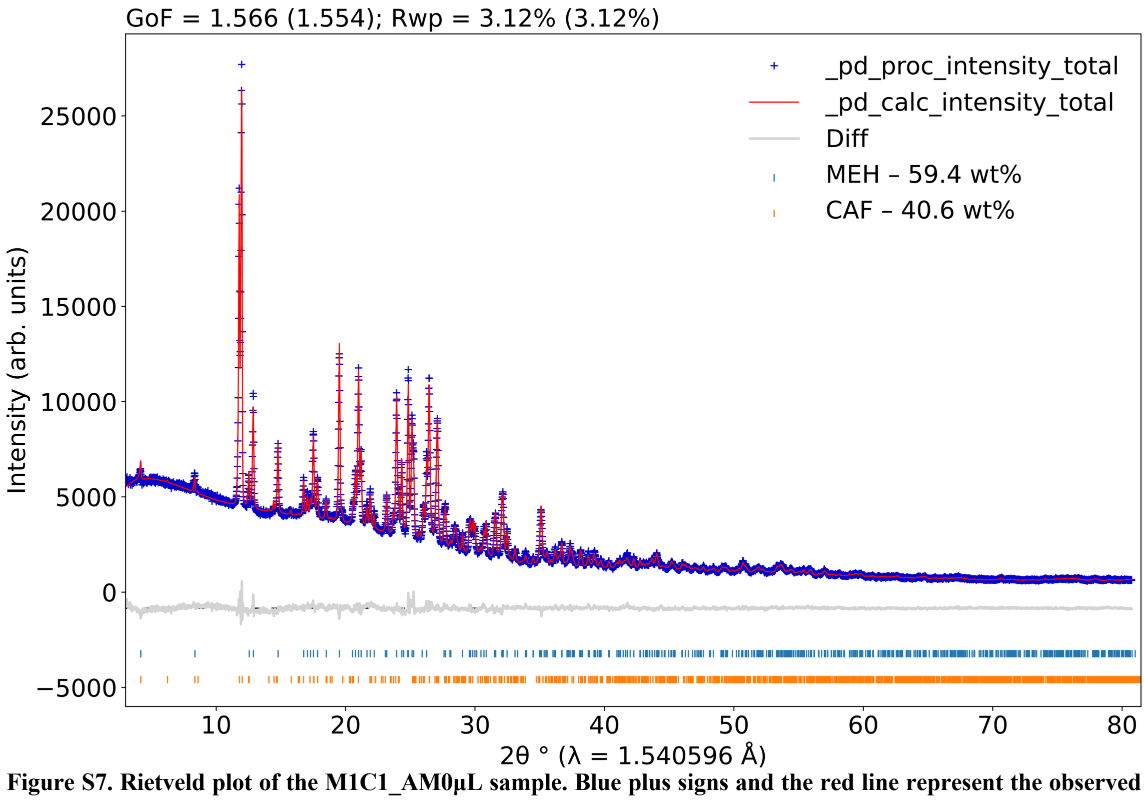

Figure S7. Rietveld plot of the M1C1_AM0μL sample. Blue plus signs and the red line represent the observed and calculated data, respectively. The grey line at the bottom indicates the difference between observed and calculated data. Blue and orange vertical bars represent the melphalan hydrochloride (MEH) and anhydrous caffeine (CAF) phases.

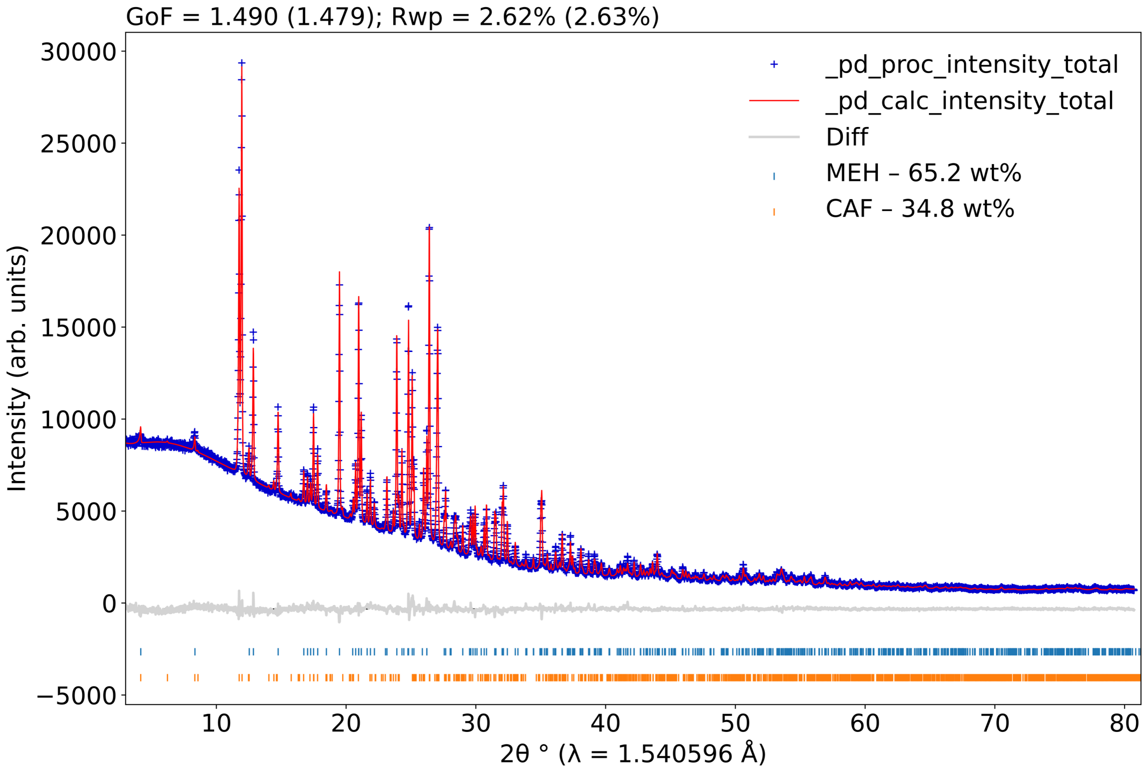

Figure S8. Rietveld plot of the M1C1_AM25μL sample. Blue plus signs and the red line represent the observed and calculated data, respectively. The grey line at the bottom indicates the difference between observed and calculated data. Blue and orange vertical bars represent the melphalan hydrochloride (MEH) and anhydrous caffeine (CAF) phases.

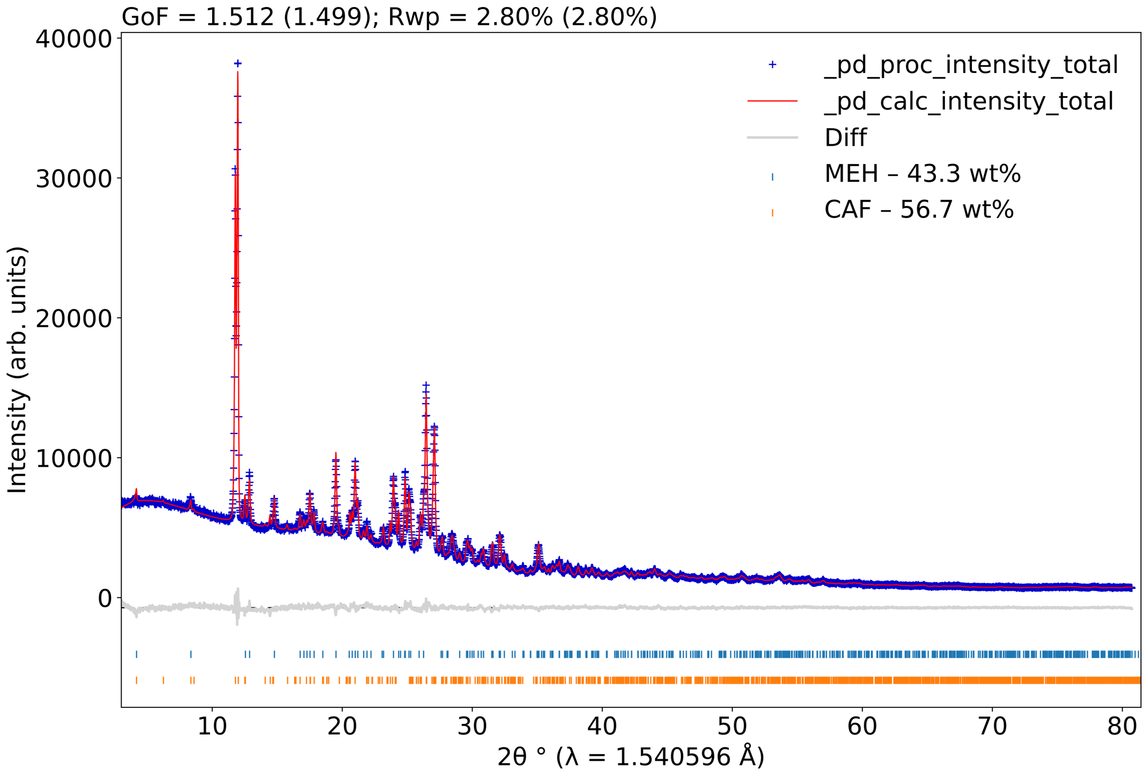

Figure S9. Rietveld plot of the M1C2_AM0µL sample. Blue plus signs and the red line represent the observed and calculated data, respectively. The grey line at the bottom indicates the difference between observed and calculated data. Blue and orange vertical bars represent the melphalan hydrochloride (MEH) and anhydrous caffeine (CAF) phases.

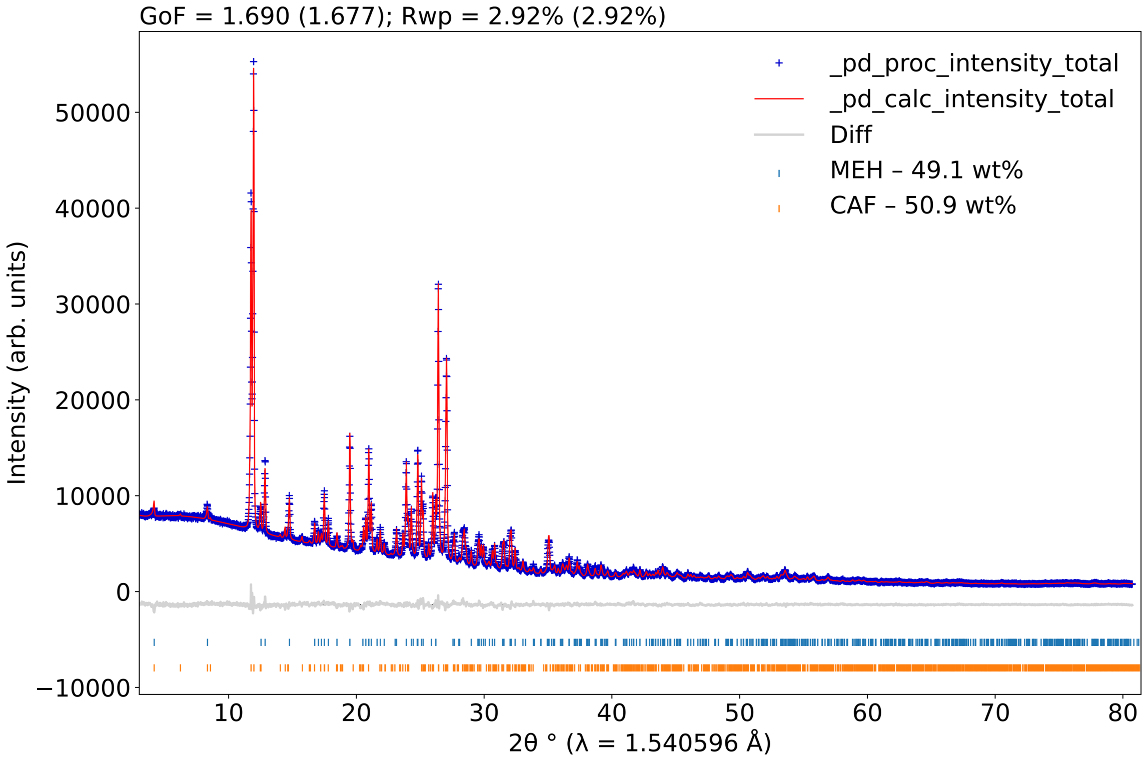

Figure S10. Rietveld plot of the M1C2_AM25µL sample. Blue plus signs and the red line represent the observed and calculated data, respectively. The grey line at the bottom indicates the difference between observed and calculated data. Blue and orange vertical bars represent the melphalan hydrochloride (MEH) and anhydrous caffeine (CAF) phases.

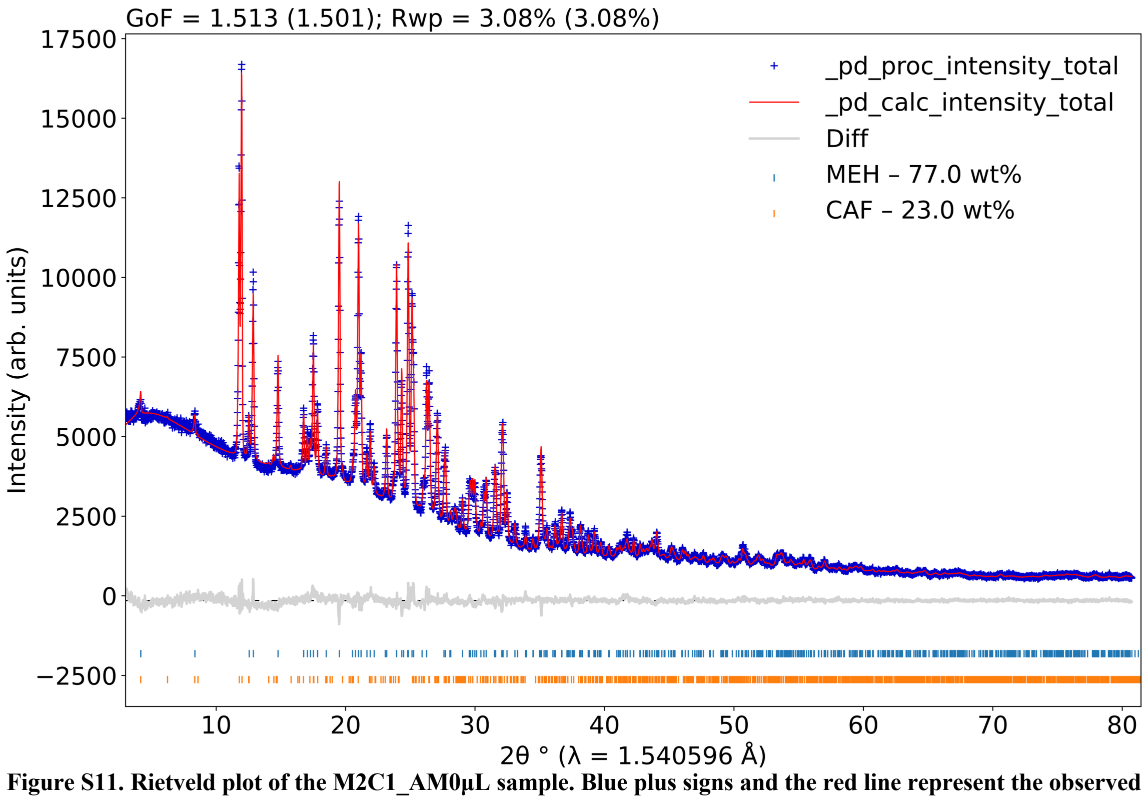

Figure S11. Rietveld plot of the M2C1_AM0µL sample. Blue plus signs and the red line represent the observed and calculated data, respectively. The grey line at the bottom indicates the difference between observed and calculated data. Blue and orange vertical bars represent the melphalan hydrochloride (MEH) and anhydrous caffeine (CAF) phases.

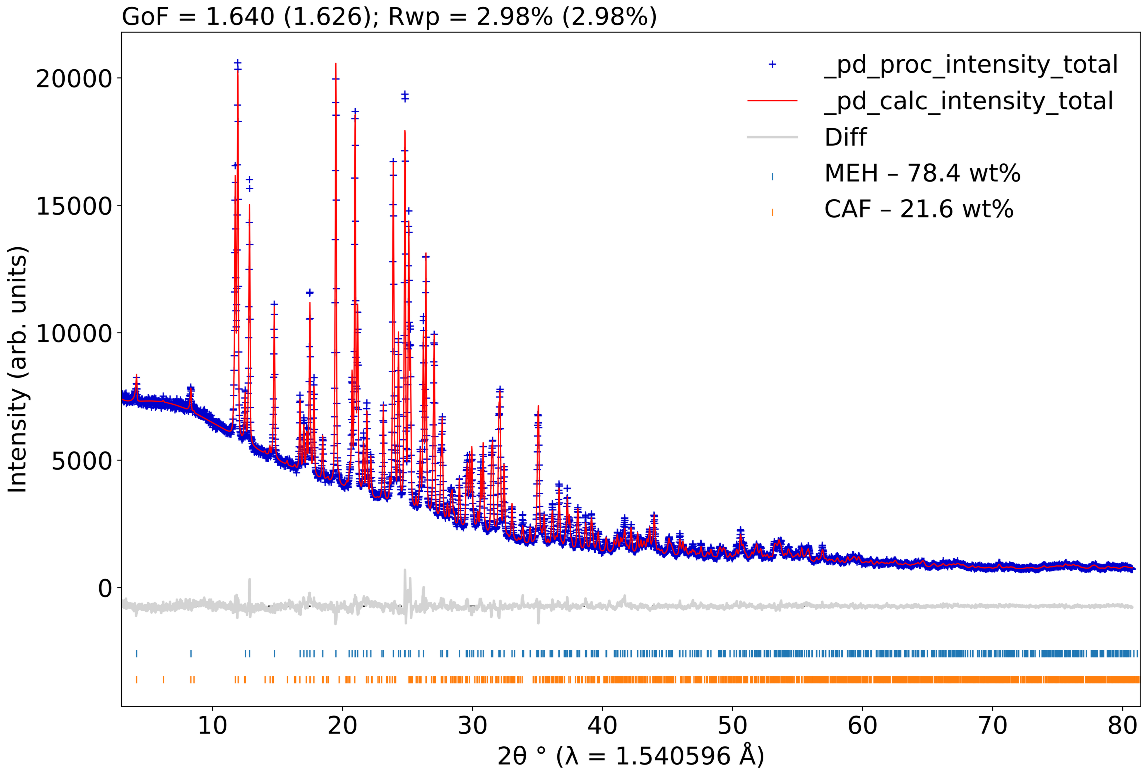

Figure S12. Rietveld plot of the M2C1_AM25µL sample. Blue plus signs and the red line represent the observed and calculated data, respectively. The grey line at the bottom indicates the difference between observed and calculated data. Blue and orange vertical bars represent the melphalan hydrochloride (MEH) and anhydrous caffeine (CAF) phases.

FTIR spectroscopy

Table S5 - Assignments for MEH, CAF, and syntheses conducted in BM and AM, in equal 1:1 ratio, both with and without the addition of water.

MEH (cm^{-1})	CAF (cm^{-1})	M1C1_AM0µL	M1C1_AM25µL	M1C1_BM0µL	M1C1_BM25µL	Assignment
	3113		3106			ν C-H
3078						ν_{asym} C-H
3032		3036		3036		ν_{sym} C-H
2972						ν_{asym} CH_2-Cl
	2955		2958			ν C=O, ν C=N, or ν C=C
2888						ν_{sym} CH_2
1734		1735	1733	1735	1735	ν_{asym} C=O
1709						ν_{sym} C=O
	1693	1695	1694	1695	1700	ν C=O, ν C=N, or ν C=C
		1655	1651	1653	1650	ν C=O
	1642					ν CH_3
1615		1613	1614	1613	1614	ν C=O
	1547					ν C=N
1522						ν_{asym} C-C
1483		1483	1482	1483	1485	δ CH_2
	1479					ν CH_2
1458						δ CH_2
	1454	1455	1455	1455	1453	δ_{asym} CH, δ OH
1444		1440				δ H-O-C
	1424	1424	1429	1429	1430	ν C=C
1395		1394	1395	1394	1394	δ H-C-N Aromatic
1363						δ C-C-H Aromatic
1358						δ C-N Aromatic
	1354	1359	1358	1359	1358	δ_{sym} CH, δ OH
1342		1341	1341	1341	1341	δ C-C-N
1297		1297				δ H-C-C-N Aromatic
1283		1281	1283	1283	1286	δ H-C-C-N Aromatic
	1231					ν CN (ring)
1220		1222	1226	1221	1223	ν C-C Aromatic
1209		1210		1208		ν C-C Aromatic
	1186					ν CO (ring)
1177		1175	1175	1176	1176	ν O-H
1131		1134	1133	1134	1132	ν H-N-C
1093		1–95		1095	1095	ν H-C-N, δ CH_2
	1071					ν CO (ring)
1051		1049	1049	1049	1059	δ H-C-Cl
	1023	1024	1024	1024	1024	ν C-C
1008						δ C-C-C
970	971		971	970	971	δ N-C
928		924	924	924	924	δ H-O-C
	860					δ CH
857		858	858	858	858	ν C-C
801		804	803	804	803	δ H-C-CN Aromatic
759		760	760	760	760	δ H-C-H
	740	739				δ CH
738		739				ν C-Cl
713		713	713	713	713	ν C-Cl
682		685	685	685	685	ν C-C Aromatic
657		657	657	657	650	δ C-C-C
506		510	510	509	509	δ C-C-N, δ N-C-C-C
	484	480	480	480	479	ν NC
432						δ N-C-C

Symbols used: δ, angular deformation; ν, stretch.

Table S6 - Attribution for MEH, CAF, and syntheses conducted in BM and AA, in 1:2 ratios, both with and without the addition of water.

MEH (cm^{-1})	CAF (cm^{-1})	M1C2_AM0µL	M1C2_AM25µL	M1C2_BM0µL	M1C2_BM25µL	Assignment
					3353	s_{sym} O–H
	3113				3119	ν CH
			3102	3102		ν O–H
3078		3092				ν_{asym} C-H
3032						ν_{sym} C-H
2972		2967	2961	2961	2964	ν_{asym} CH_2-Cl
	2955					ν C=O, ν C=N, or ν C=C
2888						ν_{sym} CH_2
1734		1735	1735	1736	1735	ν_{asym} C=O
1709					1706	ν_{sym} C=O
	1693	1695	1695	1695		ν C=O, ν C=N, or ν C=C
	1642	1648	1649	1650	1650	ν CH_3
1615		1614	1614	1614	1613	ν C=O
	1547	1546	1546	1545	1548	ν C=N
1522		1520	1520	1519	1519	ν_{asym} C-C
1483		1483	1483	1483	1484	δ CH_2
	1479					ν CH_2
1458		1455	1455	1456	1452	δ CH_2
	1454	1455	1455	1456	1452	δ_{asym} CH, δ OH
1444						δ H-O-C
	1424	1428	1428	1429	1432	ν C=C
1395		1395	1395	1395	1394	δ H-C-N Aromatic
1363						δ C-C-H Aromatic
1358		1357	1358	1359	1359	δ C-N Aromatic
	1354					δ_{sym}CH, δ OH
1342		1341	1344	1343	1342	δ C-C-N
1297						δ H-C-C-N Aromatic
1283		1283	1283	1283	1286	δ H-C-C-N Aromatic
	1231					ν CN (ring)
1220		1223	1225	1224	1222	ν C-C Aromatic
1209						ν C-C Aromatic
	1186					ν CO (ring)
1177		1176	1176	1176	1176	ν O-H
1131		1133	1133	1134	1133	ν H-N-C
1093		1094	1094	1096	1094	ν H-C-N, δ CH_2
	1071	1176	1176	1176	1176	ν CO (ring)
1051		1049	1049	1048	1049	δ H-C-Cl
	1023	1023	1023	1025	1025	ν C-C
1008						δ C-C-C
970		969	970	970	970	δ N-C
928		924	924	924	924	δ H-O-C
	860					δ CH
857		858	858	858	857	ν C-C
801		804	805	805	804	δ H-C-CN Aromatic
759		760	760	759	760	δ H-C-H
	740	741	741	741	741	δ CH
738						ν C-Cl
713		712	711	712		ν C-Cl
682		685	686	685	685	ν C-C Aromatic
657		657	657	657	652	δ C-C-C
506		510	510	510	510	δ C-C-N, δ N-C-C-C
	484	480	480	480	480	ν NC
432						δ N-C-C

Symbols used: δ, angular deformation; ν, stretch.

Table S7 - Assignments for MEH, CAF, and syntheses conducted in BM and AM, in 2:1 ratio, both with and without the addition of water.

MEH (cm^{-1})	CAF (cm^{-1})	M2C1_AM0µL	M2C1_AM25µL	M2C1_BM0µL	M2C1_BM25µL	Assignment
					3350	s_{sym} O–H
	3113				3119	ν CH
3078						ν_{asym} C-H
3032						ν_{sym} C-H
2972				2971		α_{asym} CH_2-Cl
	2955					ν C=O, ν C=N, or ν C=C
2888						ν_{sym} CH_2
1734		1735	1735	1735	1733	ν_{asym} C=O
1709					1708	ν_{sym} C=O
	1693	1679	1697	1697		ν C=O, ν C=N, or ν C=C
		1656	1656	1657	1652	s C=O
	1642					ν CH_3
1615		1613	1613	1612	1612	ν C=O
	1547	1546	1547	1547	1548	ν C=N
1522		1520	1519	1519	1518	ν_{asym} C-C
1483		1483	1483	1583	1483	δ CH_2
	1479					ν CH_2
1458		1456	1455	1455	1455	δ CH_2
	1454	1456	1455	1455	1455	δ_{asym} CH, δ OH
1444		1440	1440	1442		δ H-O-C
	1424					ν C=C
1395		1394	1394	1394	1394	δ H-C-N Aromatic
1363		1360	1360			δ C-C-H Aromatic
1358						δ C-N Aromatic
	1354					δ_{sym} CH, δ OH
1342		1341	1341	1341	1341	δ C-C-N
1297		1296	1296	1297	1294	δ H-C-C-N Aromatic
1283		1281	1281	1281	1281	δ H-C-C-N Aromatic
	1231					ν CN (ring)
1220		1223	1223	1222	1221	ν C-C Aromatic
1209		1208	1207			ν C-C Aromatic
	1186					ν CO (ring)
1177		1176	1175	1176	1174	ν O-H
1131		1133	1133	1132	1131	ν H-N-C
1093		1094	1094	1094	1094	ν H-C-N, δ CH_2
	1071					ν CO (ring)
1051		1048	1048	1049	1048	δ H-C-Cl
	1023	1025	1025	1025	1025	ν C-C
1008						δ C-C-C
970						δ N-C
928		925	924	925	924	δ H-O-C
	860					δ CH
857		858	858	858	857	ν C-C
801		804	804	804	801	δ H-C-CN Aromatic
759		760	760	761	760	δ H-C-H
	740					δ CH
738		739	739	739	738	ν C-Cl
713		712	714	714	712	ν C-Cl
682		685	685	685	684	ν C-C Aromatic
657		657	655	655	654	δ C-C-C
506		510	510	510	508	δ C-C-N, δ N-C-C-C
	484	480	481	481	479	ν NC
432		432	432			δ N-C-C

Symbols used: δ, angular deformation; ν, stretch.

UV-Visible Spectroscopy

A UV/Vis spectrophotometer (BEL Engineering, model UV-M51) was used to perform a spectral scan of 1.17 mM MEH and 2.57 mM CAF solutions at room temperature. Considering the values obtained in the literature for both molecules, the scan was performed between 244 and 301 nm with a spectral width of 2 nm. Spectral scans show absorption maxima at 261 nm for MEH and 272 nm for CAF, respectively (Figure S13a).

Considering that it was possible to observe a spectral difference between the MEH and CAF absorption maxima of 11 nm, MEH:CAF solutions comprised of 1:1 (2.65 mmol MEH and 2.65 mmol CAF), 2:1 (5.30 mmol MEH and 2.65 mmol CAF), and 1:2 (2.65 mmol MEH and 5.30 mmol CAF) were performed. The samples were analyzed the same way as the pore compound solutions, and maximum intermediate values between 261 nm and 272 nm were obtained depending on the concentrations of the components (Figure S13b). Based on what has been observed, it can be concluded that it is impossible to directly determine the concentrations of MEH and CAF of the mixtures from the absorption maxima. On the other hand, the shift from the maximum wavelengths of pure MEH and CAF to intermediate wavelengths could suggest the existence of interactions between both molecules, which could depend on their concentrations.

The conventional MEH solution allowed the visual identification of the interference peak at 227 nm and 337 nm wavelengths. The CAF was also diluted in water, and its observed spectrum was within the 244−301 nm wavelength range under ambient conditions. Consequently, the spectral intensity decreases to zero when the wavelength exceeds 301 nm. On the other hand, an additional absorbance peak becomes discernible at a wavelength below 244 nm. The spectrum of MEH was then analyzed, leading to the observation of the maximum value of λ_{max} = 261 nm with an absorbance of A = 0.86. The maximum absorbance of the CAF recorded was A = 0.98, occurring precisely at the maximum wavelength λ_{max} = 272 nm.

Three solutions were made in different proportions between MEH and CAF to discern their maximum wavelengths. The M1C1 solution, in a 1:1 ratio, presented a wavelength between 237−337 nm; the maximum wavelength observed was in λ_{max} = 265 nm, accompanied by an absorbance of A = 0.79. A second 1:2 ratio M1C2 solution exhibited a wavelength range of 240−335 nm, where the longest wavelength observed was λ_{max} = 267 nm and the absorbance value A = 0.85. The third M2C1 solution of 2:1

ratio resulted in a wavelength range ranging from 232−338 nm, with wavelength recorded at λ_{max} = 263 nm, exhibiting an absorbance value of A = 0.73.

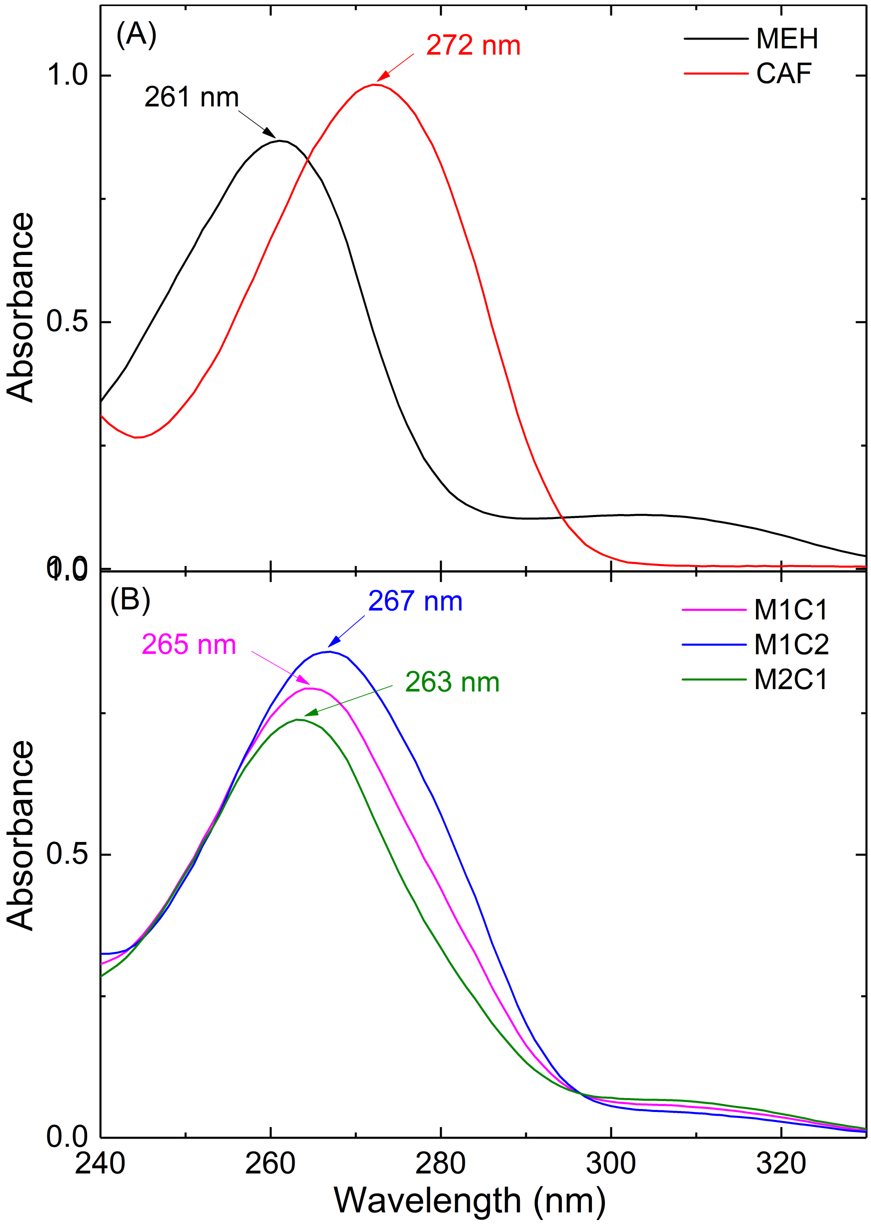

Figure S13. (a) UV-vis spectrum of MEH (black) and CAF (red); (b) UV-vis spectra comparative between MEH samples with CAF and associations: M1C1 (magenta), M1C2 (blue), and M2C1 (green).
